# Active learning meets metadynamics: automated workflow for reactive machine learning interatomic potentials

**DOI:** 10.1039/d5dd00261c

**Published:** 2025-10-30

**Authors:** Valdas Vitartas, Hanwen Zhang, Veronika Juraskova, Tristan Johnston-Wood, Fernanda Duarte

**Affiliations:** a Chemistry Research Laboratory 12 Mansfield Road Oxford OX1 3TA UK fernanda.duartegonzalez@chem.ox.ac.uk

## Abstract

Atomistic simulations driven by machine-learned interatomic potentials (MLIPs) are a cost-effective alternative to *ab initio* molecular dynamics (AIMD). Yet, their broad applicability in reaction modelling remains hindered, in part, by the need for large training datasets that adequately sample the relevant potential energy surface, including high-energy transition state (TS) regions. To optimise dataset generation and extend the use of MLIPs for reaction modelling, we present a data-efficient and fully automated workflow for MLIP training that requires only a small number (typically five to ten) of initial configurations and no prior knowledge of the TS. The approach combines automated active learning with well-tempered metadynamics to iteratively and selectively explore chemically relevant regions of configuration space. Using data-efficient architectures, such as the linear Atomic Cluster Expansion, we illustrate the performance of this strategy in various organic reactions where the environment is described at different levels, including the S_N_2 reaction between fluoride and chloromethane in implicit water, the methyl shift of 2,2-dimethylisoindene in the gas phase, and a glycosylation reaction in explicit dichloromethane solution, where competitive pathways exist. The proposed training strategy yields accurate and stable MLIPs for all three cases, highlighting its versatility for modelling reactive processes.

## Abstract 2

Automated active learning integrated with enhanced sampling facilitates data-efficient training of machine learning interatomic potentials for chemical reactions.

## Graphical abstract

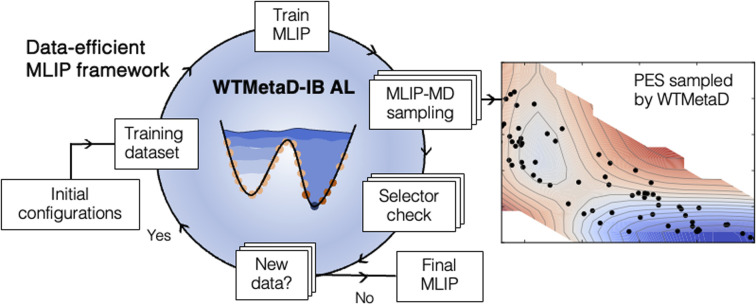

## Introduction

Understanding the mechanisms underlying chemical reactions is key in modern chemistry. Empirical observations are increasingly complemented by mechanistic insight and predictive modelling, essential for optimising synthetic procedures and discovering new molecules. Central to this goal is the adequate description of the potential energy surface (PES).^1,2^

Wavefunction and density functional theory (DFT)-based methods are well-established approaches characterising stationary points on the PES, including reactant state (RS), transition state (TS), and product state (PS). However, these static methods have limitations as, in addition to being computationally costly, they do not account for dynamics, which is essential for exploring bifurcating PESs,^3^ flexible molecules^4^ and solvent effects.^5,6^

Dynamics simulations driven by *ab initio* methods, where energies and forces are computed “on the fly” by solving the Schrödinger equation at each time step, such as *ab initio* molecular dynamics (AIMD)^7^ and quasiclassical trajectories,^8^ enable realistic modelling of reaction mechanisms. These approaches have been applied, for example, in the identification of entropic intermediates,^9^ the prediction of product ratios for reactions exhibiting bifurcating surfaces,^10,11^ and the modelling of reactions in solution.^12–14^ Despite these successes, the high computational cost of the underlying *ab initio* method limits the size, complexity, and timescale of the systems that can be studied. Moreover, these methods often require trade-offs between accuracy (level of theory used) and simulation time, leading to insufficient sampling and a failure to achieve a converged free energy surface (FES).

Machine-learned interatomic potentials (MLIPs) offer an efficient alternative to electronic structure methods used in AIMD simulations. MLIPs map a set of molecular structures to energies and, often, forces, leveraging various machine learning (ML) architectures, such as neural networks (NNs),^15,16^ graph NNs,^17,18^ kernel-based approaches,^19–21^ and linear regression techniques.^22,23^ MLIPs have found applications in a wide range of areas, including the study of organic molecules,^24^ the exploration of materials^25,26^ and the reproduction of the physical properties of bulk water.^27^ However, their capability in reaction modelling remains relatively underexplored, with only a few studies investigating the dynamics of chemical reactions, typically in the gas phase and implicit solvent, such as pericyclic^28,29^ and photochemical reactions.^30,31^ Examples of organic reactions modelling solvent explicitly include works by the groups of Parrinello,^32^ Corminboeuf,^33^ and our recent work on modelling an S_N_2 reaction in water^34^ and a Diels–Alder reaction in water and methanol.^35^

A bottleneck in the use of MLIPs for reaction modelling is the computational cost associated with the generation of training data sets representative of the PES, including configurations in the TS regions.^36^ Common strategies for data generation include dynamics sampling through AIMD that are initiated from either QM-optimised TSs or enhanced sampling.^37,38^ Techniques such as the Nudged Elastic Band (NEB)^39^ and normal mode sampling^40^ are also used to collect representative configurations on the PES. Active learning (AL)^41,42^ can further speed up training by iteratively exploring the PES using the trained MLIP-MD. This process helps to identify under-represented regions, thereby improving the performance of the MLIP while minimising structural redundancy.

Several research groups have combined AL with other strategies to sample high-energy configurations. For example, Meuwly *et al.* used AL based on a query by a committee at 1000 K to train MLIPs for a set of Diels–Alder^43^ and hydrogen transfer reactions in the gas phase.^44^ Bombarelli *et al.* integrated AL and NEB driven by MLIP to iteratively explore the PES of several organic reactions.^29^ Our group has employed AL coupled to MLIP-MD downhill sampling from an optimised TS structure to model reactions in the gas phase, implicit, and explicit solvent.^28,34,35^ While acknowledging these successes, generating training data remains challenging, in particular for systems involving flexible molecules or explicit solvent, where multiple local minima may be populated at the temperature of interest.

Recent efforts toward the development of reactive MLIPs have integrated metadynamics,^45^ or their variants,^46,47^ with AL.^48–51^ These include reactions in the gas phase, such as Diels–Alder reaction,^52^ and explicit solvent (urea decomposition in water,^32^ oxygen reduction at Au-water interfaces,^53^ ring opening of *N*-enoxyphthalimide,^33^ S_N_2 reaction,^52^ phosphoester bond formation and breaking,^49^ peptide bond formation,^50^ and a Menshutkin reaction).^54^ While these studies have significantly improved the quality and efficiency of the generated potential compared to early approaches, they still rely on extensive preliminary AIMD data, incurring significant computational costs in the early stages, which is prohibitive for large systems.

Approaches aimed to remove the need for predefined CVs in metadynamics have employed uncertainty-driven enhanced sampling techniques. Using this approach, MLIPs have been trained for modelling alloys and polymers,^55^ glycine and alanine dipeptide, metal–organic frameworks, as well as the proton transfer reaction in acetylacetone.^56,57^ While these techniques have been shown to improve the accuracy and stability of the generated MLIPs by sampling regions with large uncertainty, their effectiveness is still dependent on the careful tuning of biasing parameters,^57^ which are system-dependent and influenced by factors such as energy barrier heights and interatomic forces.

In this work, we integrate our previously reported AL strategy^28,35^ and well-tempered metadynamics (WTMetaD) to create an automated workflow that reduces the computational cost associated with dataset generation while ensuring sufficient sampling, thereby eliminating the need for prior AIMD simulations. Furthermore, we extend our strategy to include inherited bias well-tempered metadynamics (WTMetaD-IB), allowing us to carry forward the accumulated bias from previous AL iterations, and further increase the efficiency of the training process. This approach parallels the recently published incremental learning scheme applied in metal–organic frameworks.^58^ However, it directly incorporates the AL framework to enable the training of MLIP for flexible systems.

We demonstrate the performance of the WTMetaD-IB AL approach using linear Atomic Cluster Expansion (ACE) potentials^22^ to model three organic reactions, namely the S_N_2 reaction between fluoride and chloromethane in implicit water (R1), the methyl shift of 2,2-dimethylisoindene in the gas phase (R2), and the glycosylation reaction between glucosyl α-trichloroacetate and *i*-PrOH in explicit dichloromethane (DCM) solvent (R3). For the first two examples, we compare the accuracy and sampling efficiency against our previously reported AL + downhill dynamics strategy.^28,34,35^ We show that the integration of WTMetaD-IB and AL results in accurate and data-efficient MLIPs, without requiring *a priori* knowledge of the relevant TSs and/or reaction pathways. The accuracy and stability of the MLIP trained using WTMetaD-IB AL demonstrate the broad applicability of the proposed method, reducing computational cost and human intervention and facilitating the widespread use of MLIPs in modelling reactivity.

## Methodology

**Fig. 1:**
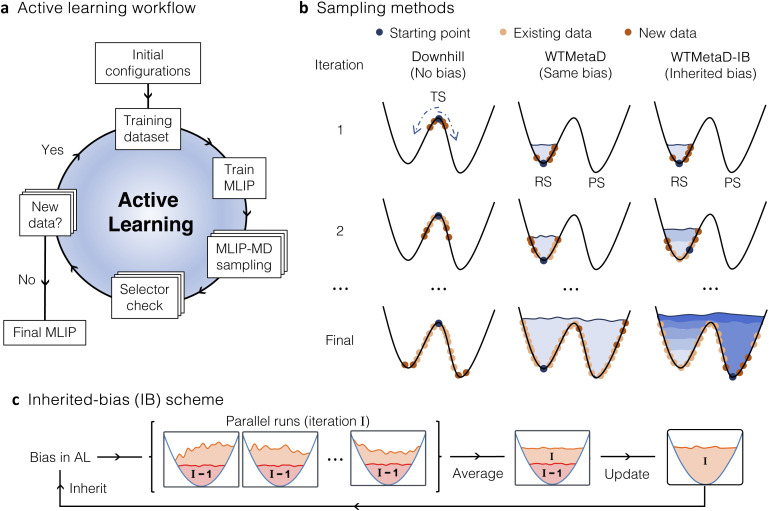
(a) Schematic representation of the Active Learning (AL) strategy for training a Machine Learning Interatomic Potential (MLIP). (b) MLIP-MD sampling can be performed using either downhill, well-tempered metadynamics (WTMetaD) or WTMetaD with inherited bias (WTMetaD-IB). Dark blue points denote the starting points in MLIP-MD, yellow points indicate the existing training data and dark orange points denote the training points selected in each iteration. The different blue shades shown in WTMetaD-IB indicate that the biases are updated after each iteration, while in WTMetaD the same bias is maintained. (c) Illustration of the inherited bias scheme used to update the bias in WTMetaD-IB AL. The bias from the previous iteration (highlighted in pink) is used as the initial bias in this iteration, while the bias for the next AL iteration is the averaged bias across the parallel runs in this iteration.

The workflow presented here builds on our previous work on an automated AL strategy for reactive MLIPs (Fig. 1a).^28,35^ The AL cycle starts with a training set of around five to ten configurations. These configurations can be obtained by a random displacement of atoms from an input structure, *e.g.*, RS or optimised TS in the gas phase or implicit solvent, or by generating a solvated cluster where solvent molecules are placed around the solute. The structures are then labelled with energies and forces computed at the ground-truth level of theory and used for training the initial version of MLIP. Subsequently, the potential is used to propagate several independent MLIP-MD trajectories in parallel for *n*^3^ + 2 fs, where *n* is the index of the MD run in the AL loop, starting from zero.

The last frame from each MLIP-MD trajectory is evaluated by a chosen selector to determine whether the structure will be added to the training set or not. If the frame is not selected, the index *n* is incremented, and the MD simulation continues. The cubic increase in simulation time as (*n*^3^ + 2) ensures gradual and continuous exploration of the PES, sampling more frequently regions near the starting point and promoting rapid exploration of the PES as the potential becomes more stable. Different scaling with *n* may also be suitable, although it may lead to slower exploration of the PES or generate too distorted configurations during MLIP-MD. The automated process iterates until the MD simulation reaches the maximum time (1 ps by default) or the maximum number of AL iterations, with a default value of 50. The resulting MLIP is then considered final, and its performance is validated on testing data sets generated independently.

Exploration of the PES can be performed by MLIP-MD using downhill dynamics, WTMetaD, or WTMetaD-IB during the AL (Fig. 1b). In the first case, training starts from a predefined TS, represented by the stationary dark blue points in the left panel of Fig. 1b. Random displacements around this point provide the initial configurations for the first version of the MLIP. Each iteration involves propagating downhill MLIP-MD simulations towards either the RS or PS, with the direction determined by the randomly assigned initial velocities.

Contrary to downhill sampling, WTMetaD can start from any point on the PES without prior knowledge of the TS geometry, making it applicable to a wider range of scenarios. However, it still requires careful selection of collective variables (CVs) that accurately represent potentially relevant pathways. For WTMetaD simulations in the AL process, energy barriers between minima are overcome by depositing Gaussians on the PES, resulting in a bias potential as indicated by the light blue regions in the middle panel of Fig. 1b. These biases are introduced along CVs, such as the lengths of breaking and forming bonds specific to the reaction. In WTMetaD, the height of the deposited Gaussians decreases with time to ensure smooth convergence of the free energy surface.^47^ Combining this with AL provides additional stability to the sampling, as it prevents the accumulation of large biases which could otherwise impede dynamics across the reaction profile.

To increase the efficiency of the AL loop and avoid redepositing identical Gaussians at approximately the same positions of the PES at the beginning of each AL iteration, we introduce the WTMetaD-IB approach, employing an inherited bias scheme (Fig. 1c). In WTMetaD-IB, the bias potential generated from the previous iteration (I-1, pink region in Fig. 1c) is carried forward to the current iteration (I). During this iteration, the new WTMetaD Gaussians are deposited atop the existing inherited potential, ensuring a varying exploration of PES in each AL iteration (right panel, Fig. 1b). When *n* MLIP-MD simulation runs with WTMetaD-enhanced bias are executed in parallel, the bias potentials produced in each trajectory (light orange, Fig. 1c) are summed up, with the height of each potential scaled by 1/*n*. This approach yields a smooth average bias that serves as the initial bias for all the trajectories in the subsequent AL iteration.

This smoothing effect is beneficial as it further mitigates the artificial roughness of the potential caused by the frequent deposition of steep Gaussians. Additionally, the starting point of MLIP-MD in each iteration is updated based on the lowest biased energy (DFT energy + inherited bias energy) among the points in the current dataset to prioritise unexplored regions (right panel, Fig. 1b). By introducing the inherited bias scheme using the updated starting points, our sampling method demonstrates increased efficiency relative to WTMetaD sampling. A comparative analysis of WTMetaD and WTMetaD-IB is presented below.

## Results and discussion

### WTMetaD-IB AL *vs.* downhill AL – S_N_2 reaction as case study

As an initial step, we compare the performance of the combined WTMetaD-IB AL strategy against downhill AL, using the reaction between the fluoride ion and chloromethane (R1) as a model system. The MLIPs were trained at the CPCM(water)-PBE0-D3BJ/def2-SVP level of theory. While this study uses a relatively simple hybrid functional to compute the reference data, the same procedure could be applied with more accurate electronic structure methods. For WTMetaD-IB, the difference between two distances, C–Cl and C–F (*r*_Cl_ − *r*_F_) was used as CV, while for downhill dynamics a TS, optimised at the ground-truth level of theory, was used to generate initial training data.

**Fig. 2:**
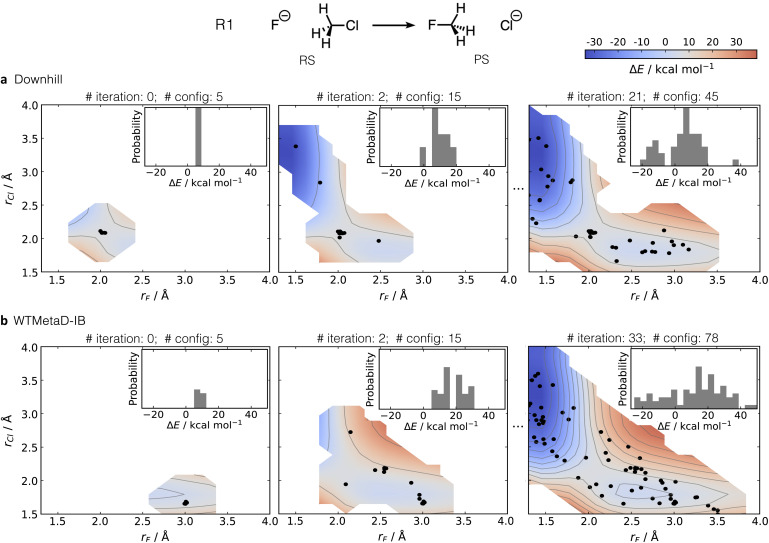
MLIP training for the S_N_2 reaction between fluoride and chloromethane in implicit water using a downhill and (b) WTMetaD-IB for AL sampling. The number of AL iterations and configurations generated is listed at the top of each sub-plot. Black dots represent training data collected during the AL iterations. The illustrative 2D PESs as a function of the forming (*r*_F_)/breaking (*r*_Cl_) bonds were generated by a relaxed potential energy scan using the MLIP. The probability density (ranging from 0.00 to 0.06) of energy on the generated data points during training is shown in the inner plots relative to the optimised RS.

**Fig. 3:**
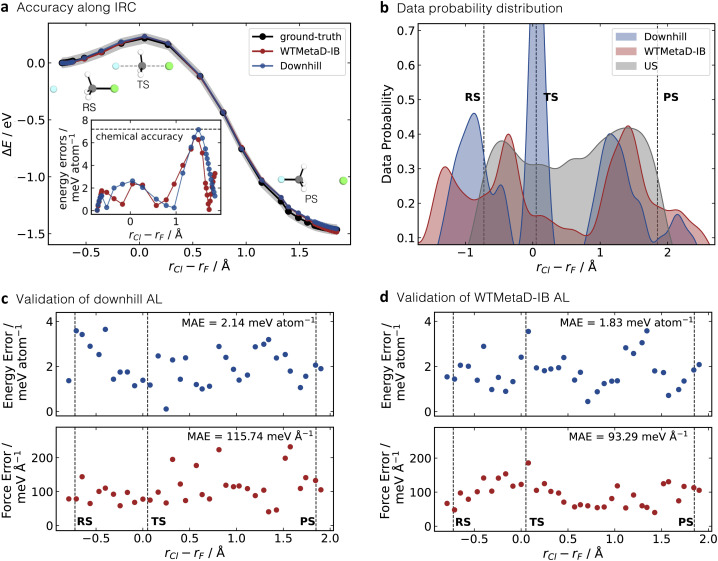
(a) Point-to-point energy validation of two MLIPs trained by downhill and WTMetaD-IB AL. Data points were generated from an Intrinsic Reaction Coordinate (IRC) calculation and plotted here along the *r*_Cl_ − *r*_F_ coordinate. The shaded region bounds the 1 kcal mol^−1^ area of accuracy. The predicted energy errors are shown in the inner plot at the bottom left. (b) Data distribution of training data for downhill (45 points, blue), WTMetaD-IB (78 points, red) and testing data (322 data points, grey) generated from a short umbrella sampling AIMD simulation (US/AIMD). Energy (upper panel) and force (bottom panel) errors on the US/AIMD test set using the MLIPs trained *via* (c) downhill and (d) WTMetaD-IB AL. Points represent the mean absolute error (MAE) for test points within each of the 40 equally spaced intervals, ranging from −1 to 2 along the *r*_Cl_ − *r*_F_ reaction coordinate. The positions of RS, TS and PS are highlighted by dashed lines.

Fig. 2a shows the gradual exploration of the PES for this reaction, with the training configurations depicted as black dots. Downhill AL starts from a series of structures generated by random displacement of the DFT-optimised TS (AL iteration 0). These structures are located in the high-energy region of the PES (inner plot). In contrast, WTMetaD-IB AL begins with distorted (not optimised) RS configurations, as evidenced by the extended C–F bond and energies comparable to TS configurations (see Fig. 2 and 3a).

In subsequent iterations, both approaches successfully sample the PES, as shown in the right panel of Fig. 2a and b. The AL process consisted of 21 iterations for downhill AL and 33 iterations for WTMetaD-IB AL, resulting in 45 and 78 training points, respectively. The fewer iterations required for downhill sampling are due to its ability to sample both RS and PS from previously calculated TS within a single iteration (Fig. 2a), facilitated by propagating such dynamics at high temperature (500 K). In contrast, WTMetaD-IB AL, conducted at a lower temperature (300 K), explores the reaction space in a more unidirectional manner. Despite this, WTMetaD-IB AL provides a more uniform distribution of energies in the training data, resulting in better accuracy of the MLIPs in both energies and forces.

The accuracy of the MLIPs was evaluated by comparing the predicted energies with those obtained from the ground-truth method (CPCM(water)-PBE0-D3BJ/def2-SVP) along the intrinsic reaction coordinate (IRC, Fig. 3a) obtained from DFT calculations. The mean absolute error (MAE) in the energy for the trained MLIPs is 2.66 meV atom^−1^ for downhill AL and 2.12 meV atom^−1^ for WTMetaD-IB AL. Both values are within chemical accuracy (1 kcal, equating to 43 meV and 7.17 meV atom^−1^ for this system). The predicted energy errors for each configuration in the IRC are depicted in the subplot of Fig. 3a. The individual predicted energy errors for both MLIPs are below 1 kcal mol^−1^ as well, except for one data point predicted by MLIP trained using the downhill AL with an error of 7.17 meV atom^−1^ (1 kcal mol^−1^). Interestingly, while the TS optimised by the ground-truth method was not provided in WTMetaD-IB AL, the MLIP trained using this method still reached a higher accuracy for the TS with an error of 2.37 meV atom^−1^ in comparison to 2.63 meV atom^−1^ by downhill sampling. This shows the applicability of the WTMetaD AL in sampling the region close to TS and the ability of the resulting MLIP to predict the energy of the TS.

We evaluated the quality of the trained MLIPs using an independent test set obtained from a 3.2 ps umbrella sampling (US) simulation at the ground-truth level of theory (US/AIMD). This simulation produced 322 testing data points collected every 10 fs. Fig. 3b depicts the probability distributions of the training data sampled along the reaction coordinate *r*_Cl_ − *r*_F_ for downhill AL (blue), WTMetaD-IB AL (red), in comparison with the testing US data (grey). This analysis indicates that training data generated *via* downhill AL is concentrated in the TS region, with clear gaps between the RS and PS regions. The high probability of structures near the TS in downhill AL is influenced by the selection of the five initial configurations encompassing the TS and its randomly displaced geometries. On the other hand, the data collected using WTMetaD-IB AL does not show any significant gaps, further confirming that it leads to a more uniform sampling of the PES.

When comparing downhill AL and WTMetaD-IB AL, WTMetaD-IB AL shows slightly lower energy and force errors (1.83 meV atom^−1^ and 93.29 meV Å^−1^, respectively) compared to downhill AL (2.14 meV atom^−1^ and 115.74 meV Å^−1^), as depicted at Fig. 3c and d. The marginally better performance for WTMetaD-IB can be attributed to its more uniform sampling across the energy and reaction coordinate space (Fig. 3b). Downhill AL demonstrates higher accuracy around the TS region, defined as configurations with *r*_Cl_ − *r*_F_ ±0.1 Å from the optimised TS (dashed line in Fig. 3c and d), with energy and force errors of 1.71 meV atom^−1^ and 74.99 meV Å^−1^, respectively.

The PS region, similarly defined by the deviation from the optimised PS (dashed line in Fig. 3c and d) within ±0.1 Å, shows the largest energy prediction error along the IRC for both methods (Fig. 3a). However, these inaccuracies are not observed in the testing data generated from US/AIMD simulations. The contrasting MLIP performance on the two test sets arises from the different configurations generated by IRC and US/AIMD, where the former presents an idealised scenario, with the Cl–C–F bond angle remaining almost constant at 180° during the reaction, while during US/AIMD, it ranges from 150° to 180° illustrating the dynamic nature of the reactive processes (Fig. S3). In addition to the accuracy validation across the PES of interest, the stability of the resulting MLIPs, employing both downhill and WTMetaD-IB sampling methods, was evaluated using 100 ps of MLIP-MD simulations under the NVE ensemble (see Fig. S4). The total energies remained constant throughout the 100-ps simulation, indicating that both sampling techniques yield stable MLIPs.

In summary, while WTMetaD-IB AL requires more iterations than downhill AL, its ability to generate uniformly represented training sets results in an overall better accuracy of the resulting MLIP at only a small additional computational cost compared to downhill AL. For example, for this reaction, WTMetaD-IB AL consumes only about 10 CPU hours more than downhill AL (240 *vs.* 230 CPU hours in total), which is largely balanced by the time required for TS optimisation. However, this come at the cost of slightly reduced accuracy in the TS region, where downhill sampling performs better due to the explicit inclusion of near-TS configurations in the training data, as summarised in Table S4.

We further compared the performance of WTMetaD-IB and standard WTMetaD during AL for MLIP training. WTMetaD required 46 iterations and generated 130 training configurations, with a total computational cost of 462 CPU hours. In contrast, WTMetaD-IB required only 33 iterations and 78 configurations, using 240 CPU hours—nearly half in both data volume and compute time. Despite using fewer configurations, WTMetaD-IB achieved better MLIP accuracy than standard WTMetaD on the US/AIMD test set (listed in Table S2), with lower MAEs for energies (1.83 *vs.* 2.22 meV atom^−1^) and forces (93.29 *vs.* 119.42 meV Å^−1^). Notably, significant deviations in energy predictions were observed for the WTMetaD trained model in the RS and PS regions (9.95 meV atom^−1^ and 10.79 meV atom^−1^, respectively), as shown in Fig. S5.

The efficiency of WTMetaD-ID arises from the use of enhanced sampling with iterative bias, which steers exploration toward chemically relevant, high-uncertainty regions of the PES while avoiding repeated sampling of already well-explored areas. This reduces redundancy in the training set and enhances the diversity and relevance of sampled configurations. Overall, our results demonstrate that WTMetaD-IB substantially improves data and computational efficiency and the accuracy of the resultant MLIPs. Further details are provided in SI§S2.2.

### Free energy barriers through the WTMetaD-IB AL approach – methyl rearrangement

**Fig. 4:**
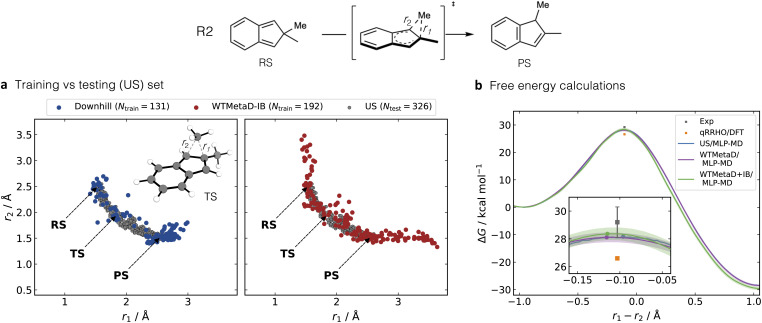
(a) Final training datasets from downhill and WTMetaD-IB sampling, and the test set obtained with a short US/AIMD simulation for reaction R2 in the gas phase. (b) Δ*G*^‡^ obtained from US, WTMetaD and WTMetaD with inherited bias (IB, WTMetaD + IB) using the MLIP trained with WTMetaD-IB AL. Shaded regions represent 95% confidence intervals derived from independent repetitions. The experimental and DFT-estimated Δ*G*^‡^, calculated using the quasi-rigid-rotor-harmonic-oscillator (qRRHO) approximation, are denoted by grey and orange squares, respectively. The bottom panel provides a zoomed-in view of the TS region, indicating the point of highest free energy, TS, in each simulation.

To evaluate the general applicability of the WTMetaD-IB AL approach to other reaction mechanisms, we studied the methyl shift of 2,2-dimethylisoindene, leading to 1,2-dimethylindene, in the gas phase (R2, Fig. 4). Rearrangement reactions play a significant role in synthetic organic chemistry,^59^ with methyl shift being a key step in the Meinwald rearrangement^60^ and the synthesis of meroterpenoids.^61^ R2 has previously been studied experimentally in pentane,^62^ reporting an activation free energy (Δ*G*^‡^) of 29.2 ± 1.1 kcal mol^−1^ at 365.6 K and suggesting a concerted mechanism based on experimental and computed thermodynamic data. Since solvent is unlikely to influence the mechanism of this reaction, MLIPs were trained only in the gas phase.

As observed for reaction R1, for R2 downhill AL also generates fewer data points (131) than WTMetaD-IB AL (192), with WTMetaD-IB AL exhibiting a more uniform sampling (Fig. S9). Furthermore, WTMetaD-IB samples longer bond lengths than umbrella sampling. Such broader sampling can help the MLIP learn physically relevant but less frequently visited configurations, particularly in high-energy regions, which can improve the robustness of the trained MLIP when encountering out-of-distribution geometries during longer MD-MLIP simulations.

The quality of the resulting MLIPs was evaluated using an independent test set from a 3 ps US/AIMD simulation at the PBE0-D3BJ/def2-SVP level of theory, with a reaction coordinate of *r*_1_ − *r*_2_, where *r*_1_ corresponds to the C–C distance between the shifted methyl group and its original position and *r*_2_ is the C–C distance between the same methyl group and its new position (upper part in Fig. 4). This simulation provided 326 testing data points, collected every 10 fs. Fig. 4a depicts the overlap between the data in the training set and test set in terms of *r*_1_ and *r*_2_ distances. The training data generated by both AL methods cover the PES explored by the test data, but WTMetaD-IB AL also includes metastable regions characterised by the formation of one C–C bond while the other C–C bond remained longer than in stable regions (points beyond the RS and PS, *i.e.*, *r*_1_ > 3 Å or *r*_2_ > 2.5 Å in Fig. 4a right panel).

On this test set, WTMetaD-IB AL performs slightly better than downhill AL, with energy and force errors of 1.49 meV atom^−1^ and 161.76 meV Å^−1^, respectively, which are 0.27 meV atom^−1^ and 13.34 meV Å^−1^ lower than those obtained using downhill AL (Table S3). The MLIP trained with WTMetaD-IB AL shows the highest errors around the TS region, defined as −0.2 Å < *r*_1_ − *r*_2_ < 0.0 Å, with the DFT TS located at *r*_1_ − *r*_2_ ≈ −0.1 Å (Fig. S9). In this region, MAE in energy and force are 4.27 meV atom^−1^ and 290.24 meV Å^−1^, respectively. The MLIP trained by downhill AL has a high error in the region between TS and PS (MAE of 4.85 meV atom^−1^ and 319.26 meV Å^−1^ for energy and force, respectively), correlating with the sampling gap between TS and PS. This suggests that the better performance of WTMetaD-IB AL arises from a more uniform sampling compared to downhill AL.

We use the MLIP generated by WTMetaD-IB to calculate the free energy of R2 along the reaction coordinate of *r*_1_ − *r*_2_ using US and WTMetaD. The US/MLIP-MD calculations used 30 windows with 40 ps per window, totalling 1.2 ns, while WTMetaD/MLIP-MD ran for 500 ps, during which the MLIP remained stable. The computed activation free energy (Δ*G*^‡^) from US/MLIP-MD was 28.2 ± 0.1 kcal mol^−1^ and 28.1 ± 0.3 kcal mol^−1^ from WTMetaD/MLIP-MD. For comparison, the Δ*G*^‡^ was computed at the ground-truth level of theory (PBE0-D3BJ/def2-SVP) using the quasi-rigid-rotor-harmonic-oscillator (qRRHO) approximation, yielding a value of 26.6 kcal mol^−1^. The dynamics results from MLIP-MD are in excellent agreement with the experimentally measured value in pentene (29.2 ± 1.1 kcal mol^−1^ at 365.6 K,^62,63^Fig. 4b), while the static DFT prediction underestimates the barrier by 2.6 ± 1.1 kcal mol^−1^. Moreover, the TS geometries obtained from both methods deviate by only 0.01 Å from the DFT-optimised TS geometry (*r*_1_ − *r*_2_ = −0.10 Å) along the reaction coordinate. These results further confirm the accuracy of the MLIP, besides the prediction of energies and forces.

The bias potential from WTMetaD-IB AL can be used to speed up the free-energy WTMetaD simulation. While this bias potential is not directly proportional to the actual free energy, it serves as a good starting point for WTMetaD.^47^ Specifically, the bias generated after the 16th iteration from WTMetaD-IB AL was used as the initial bias in the WTMetaD simulation, referred to as WTMetaD + inherited bias (WTMetaD + IB) simulation. This accelerated convergence while maintaining the accuracy of WTMetaD started from the unbiased surface (Fig. S12). The free energy barrier computed by WTMetaD + IB is 28.4 ± 0.4 kcal mol^−1^, which is consistent with standard WTMetaD (Fig. 4b). It is important to note that careful selection of the initial inherited bias is necessary to avoid potential instabilities in the dynamics. These can occur due to a large initial Gaussian height in WTMetaD combined with significant biasing potential accumulated during the AL. This scenario might push the dynamics into irrelevant high-energy regions not sufficiently sampled during the AL. A cautious approach to avoid this behaviour is to select the bias potential from the middle iteration, here iteration 16 (out of 37). However, more work is needed to provide a robust guideline on the selection of initial bias from the WTMetaD AL.

### Explicit solvation – glycosylation reaction

**Fig. 5:**
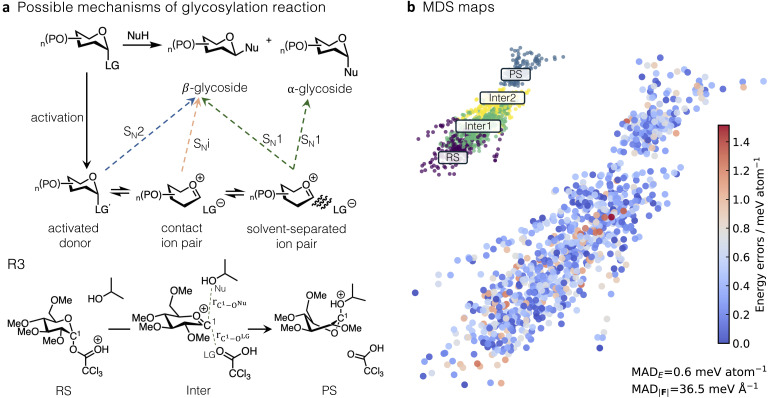
(a) Reaction pathways available in glycosylation. The specific reaction studied in this work (R3) with a possible intermediate with key bond distances highlighted is shown at the bottom. (b) (top) Multidimensional scaling (MDS) map of the SOAP descriptor centred on the anomeric atom, C^1^, for 1004 testing data points. The points are colour-coded as RS, Inter1, Inter2, and PS. Inter1 and Inter2 are determined by *r*_C^1^–O^LG^_ and *r*_C^1^–O_^:^_^Nu^_ If *r*_C^1^–O^Nu^_ > *r*_C^1^–O^LG^_ the configuration is assigned as Inter1; otherwise, it is considered Inter2 (detailed in SI§S4.1). The same MDS map is also colour-coded based on the energy error of the MLIP prediction compared to the ground truth method (ωB97X-D3BJ/def2-TZVP), with high errors in red and low errors in blue.

Having successfully applied our training strategy to R1 and R2, we extended this approach to model the acid-catalysed glycoside bond formation between glucosyl α-trichloroacetate and *i*-PrOH in explicit DCM, R3 (Fig. 5a). Controlling the stereoselectivity of glycosylation, a key step in carbohydrate synthesis, remains a significant synthetic challenge (Fig. 5a).^64,65^ This arises from the range of mechanistic pathways available, ranging from concerted S_N_2 to stepwise S_N_1-type, the latter involving a transient oxocarbenium intermediate,^66,67^ or an S_N_i pathway,^68,69^ characterised by a contact ion pair (Fig. 5a). Both experimental^70^ and computational studies^14,71^ have shown that stereoselectivity is influenced by multiple factors, including the nature of the leaving group, substituents, temperature, and solvent choice. Generally, non-polar solvents promote the S_N_2 mechanism, whereas polar solvents favour an S_N_1 mechanism by solvating the ions to form solvent-separated ion pairs.^72,73^ In DCM (dielectric constant, *ε*, of 8.93) at 223 K, the β-glycoside product is favoured, suggesting an S_N_2-like mechanism. However, this preference diminishes at 303 K, indicating a change to S_N_1-like mechanism.^64^ This behaviour further illustrates the complexity of the competition between the reaction mechanisms and the importance of an accurate description of the subtle interactions with the solvent.

Computational studies on these systems have typically employed implicit solvation models,^67,74^ overlooking the potential solvent stabilisation of intermediates. Liu and co-workers applied AIMD using the PBE-D3/DZVP level of theory to explore the reaction of a glucosyl trichloroacetimidate donor with different alcohol acceptors in explicitly modelled solvent: DCM, acetonitrile and methyl *tert*-butyl ether.^71^ Their work revealed that the preferred pathway depends on the solvent and its ability to stabilise the oxocarbenium intermediate.^71^ This agrees with the experimental study by Seeberger *et al.*,^64^ demonstrating that while the leaving groups do not significantly affect stereoselectivity, the solvent and temperatures can lead to variations in the outcome.

Here, we employ the WTMetaD-IB AL workflow to train an MLIP for modelling a reaction similar to that studied by Liu *et al.* in ref. 71 in DCM. To reduce the number of elements in the system, we replaced trichloroacetimidate leaving group with trichloroacetate. This change was motivated by the computational and memory requirements of ACE potential, which encountered out-of-memory issues when more than four elements were present. An experimental study in ref. 64 shows that the leaving group does not significantly influence the product ratio or the overall reaction mechanism.

The training dataset consists of three independent subsets generated by WTMetaD-IB AL, each designed to capture different interactions. Subset 1 consists of 154 gas-phase configurations describing the intrinsic reactivity of the system. Subset 2 consists of 245 configurations of the solute solvated with 44 DCM molecules randomly placed within an 18.5 Å box, targeting solute–solvent interactions. Finally, subset 3 includes 166 configurations of 28 DCM molecules in a 12.5 Å box, describing bulk solvent–solvent interactions. Subsets 1 and 2 were generated by WTMetaD-IB AL initiated from the RS with CV of bond length difference between *r*_C^1^–O^LG^_ and *r*_C^1^–O^Nu^_, minimising potential bias towards a specific mechanism, while subset 3 was collected without any bias. During WTMetaD-IB AL, configurations were labelled with energies and forces computed at the PBE-D3BJ/def2-TZVP level of theory, balancing computational cost and reliable prediction of structures along the reaction paths. This strategy yielded 565 configurations in total (subset 1–3). Validation of the MLIP revealed that the highest errors were located at a small number of configurations in the PS region (see Fig. S15). To improve the accuracy of MLIP in this region, the six structures with the highest errors were selected from the validation sets, the solvent molecules were removed, and the remaining gas-phase configurations were added to the training set. This improved the accuracy in the out-of-equilibrium region in PS (Fig. 5, detailed discussion is provided in SI§S4.1).

To further increase the accuracy of the predicted reaction barriers, the 571 configurations were re-labelled at the ωB97X-D3BJ/def2-TZVP level of theory, which has been shown to better describe non-covalent interactions and activation energies in organic systems.^75,76^ This re-labelled dataset was then used to train the final ACE MLIP. Further details on the training method are provided in SI§S4.1.

To prevent data leakage, the MLIPs were tested on configurations of the substrate immersed in 56 solvent molecules, representing a slightly larger system not included in the training. These configurations were generated through four independent uphill MD simulations, resulting in a total of 1004 configurations (Fig. S14); further details can be found in SI§S4.1. Visualisation of the chemical space covered by this test set, using a multidimensional scaling (MDS) map with the smooth overlap of atomic positions (SOAP) descriptor,^77^ shows that it includes configurations across the PES, including RS, intermediates, and PS (Fig. 5b). The intermediate region is divided into two subregions, Inter1 and Inter2, based on the *r*_C^1^–O^LG^_ and *r*_C^1^–O^Nu^_ distances. If *r*_C^1^–O^Nu^_ > *r*_C^1^–O^LG^_ the intermediate is classified as Inter1, structurally closer to RS; otherwise, it is considered Inter2, closer to PS. As shown in Fig. S18, no systematic trend is observed across these classes, and the error distributions are similar in width. The MAD in energy and forces between the ground truth and the MLIP-predicted values on the test set are 0.60 meV atom^−1^ and 36.50 meV Å^−1^, respectively. The maximum energy error is found in the Inter1 class (1.51 meV atom^−1^). The accuracy and absence of correlation between energy errors and configuration classes indicate that WTMetaD-IB AL collected relevant structures along the reaction R3 pathway. The MLIP thus achieves high accuracy for configurations across the whole PES of interest.

**Fig. 6:**
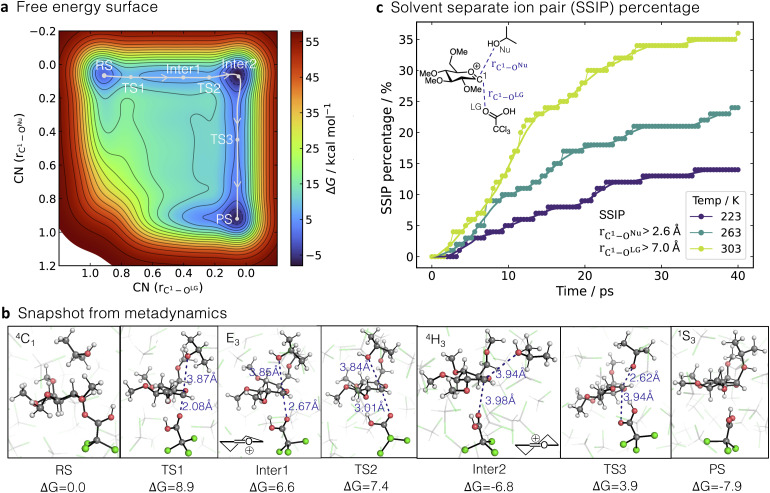
a) Free energy surface of R3 calculated using WTMetaD/MLIP-MD with ωB97X-D3BJ/def2-TZVP level of accuracy along the collective variable (CV) defined by the coordination numbers (CN): CN(*r*_C^1^–O^Nu^_) and CN(*r*_C^1^–O^LG^_), representing the breaking and forming bonds. The reaction pathway and critical points are highlighted in white. (b) Snapshots from WTMetaD simulation illustrating RS, TSs intermediates (Inter) and PS regions, highlighting *r*_C^1^–O^Nu^_ and *r*_C^1^–O^LG^_ distances. (c) Percentage of trajectories that lead to solvent-separated ion pairs (SSIP) in 100 MLIP-MD simulations initialised from Inter2 extracted from WTMetaD trajectories over 40 ps at different temperatures. SSIP is characterised by *r*_C^1^–O^Nu^_ > 2.6 Å and *r*_C^1^–O^LG^_ > 7 Å.

Using the trained MLIP at ωB97X-D3BJ/def2-TZVP level of theory, the mechanism of R3 was studied through WTMetaD, using as the collective variable (CV) the two coordination numbers (CN(*r*_C^1^–O^Nu^_) and CN(*r*_C^1^–O^LG^_)) representing the breaking and forming bonds, where a value of zero represents no bond and a value of one indicates full bond formation. The resulting FES suggests that the reaction follows a stepwise mechanism (Fig. 6a), which is consistent with the FES obtained using MLIP at the PBE-D3BJ/def2-TZVP level of theory (SI§S4.2.1). TS1 corresponds to a dissociative TS with an activation energy of 8.9 kcal mol^−1^ relative to the reactant complex. This reference, which omits the additional entropy cost of bringing the molecules together, was chosen for ease of comparison with ref. 71. TS1 adopts a chair configuration, with bond distances of 2.08 Å for *r*_C^1^–O^LG^_ and 3.87 Å for *r*_C^1^–O^Nu^_ (Fig. 6b).

Following TS1, *r*_C^1^–O^LG^_ increases further, leading to an intermediate state, Inter1. This state adopts an envelope (E_3_)-like ion conformation with *r*_C^1^–O^LG^_ of 2.67 Å. TS2 links the (E_3_)-like and half-chair (^4^H_3_)-like oxocarbenium (Inter2). Inter2 is 6.8 kcal mol^−1^ lower in energy than the RS and is stabilised by hydrogen bond interactions between the leaving group and nucleophile (Fig. S25b). Finally, TS3, corresponding to the addition of the nucleophilic group leading to the final PS,^78^ presents an energy barrier of 10.7 kcal mol^−1^ (measured from Inter2, 3.9 kcal mol^−1^ higher relative to RS) and is characterised by *r*_C^1^–O^LG^_ of 3.94 Å and *r*_C^1^–O^Nu^_ of 2.62 Å (Fig. 6b).

Our results are overall consistent with the AIMD study at the PBE-D3/DZVP level of theory from ref. 71, showing a similar reaction mechanism but with slight differences in relative energies and distances at the TSs. For example, we computed an energy barrier for TS1 of 8.9 kcal mol^−1^, while Liu *et al.* reported a value of only 1.8 kcal mol^−1^. Moreover, the computed energy for Inter2 is much lower than that of ref. 71.

The computed FES indicates that R3 occurs *via* a stepwise mechanism at 300 K; however, it does not provide sufficient information to distinguish between the S_N_i and S_N_1 pathways. The key difference between these mechanisms is the formation of a solvent-separated ion pair (SSIP) in the S_N_1 pathway, forming both the α-product and β-product (Fig. 5a). In contrast, the S_N_i mechanism maintains the contact ion pair, providing access solely to the β-product, similar to the *S*_N_2 pathway. Experimental data suggest that the mechanisms vary with temperature, as reflected by the temperature-dependent ratio of the α and β-product formation.^64^ To investigate how the mechanism changes with temperature, 100 MLIP-MD simulations were conducted at 223 K, 263 K, and 303 K, respectively (Fig. 6c). These simulations were initiated from the contact ion pair, Inter2, extracted from WTMetaD trajectories in explicit DCM and propagated for 40 ps without any bias. We monitored the formation of the SSIP, defined by the distances *r*_C^1^–O^LG^_ > 7 Å and *r*_C^1^–O^Nu^_ > 2.6 Å, where the LG is already far enough from the sugar to allow the insertion of the DCM solvent molecule, but the incoming Nu is further than the *r*_C^1^–O^Nu^_ bond distance in TS3, ensuring that the ion pair can separate while β-product has not been formed yet. As anticipated, with increasing temperature, the prevalence of the contact ion pair decreased, leading to an increase in the percentage of SSIP from 14% at 223 K to 36% at 303 K. This trend confirms the decreasing stability of the contact ion pairs in DCM as temperature increases, which agrees with experimentally observed trend of ratios of α and β-product.^64^

It is important to emphasise that we reached our conclusions without making any preliminary assumptions about the reaction mechanism. Furthermore, compared to AIMD simulation, the total time required for training and evaluating the MLIP for FES calculations is negligible, less than 0.001% of the AIMD cost, considering each femtosecond takes more than 22 hours with 8 CPUs (more details can be found in SI § 4.2.1).

## Conclusions

One of the challenges in using reactive MLIPs is the efficient acquisition of training datasets that include both minima and non-equilibrium configurations. In this study, we propose a training strategy that combines MLIP-driven WTMetaD and, optionally, inherited bias with active learning, termed WTMetaD AL and WTMetaD-IB AL, respectively. By integrating enhanced sampling with AL, we create datasets that cover the entire relevant PES, including high-energy regions, without prior knowledge of the PES.

The performance of this methodology is demonstrated across diverse organic reactions, an S_N_2 reaction between the fluoride ion and chloromethane in implicit water (R1), the methyl shift of 2,2-dimethylisoindene to 1,2-dimethylindene in the gas phase (R2) and glycosylation reaction between acid-activated glucosyl α-trichloroacetate and *i*-PrOH in explicit DCM (R3). Overall, the WTMetaD/WTMetaD-IB AL provides an efficient approach to training MLIPs for reaction modelling without prior knowledge of TSs or reaction pathways. This allows for an in-depth study of the reaction dynamics and the influence of temperature and solvent on the mechanism. While WTMetaD-IB still requires some knowledge of the system to define CVs, we envision that the use of emerging automated methods to identify CVs from limited trajectory data will further improve its efficiency.

## Computational details

### MLIPs training

MLIPs were trained using ACE.jl v0.8.4 wrapped with pyjulip *via mlp-train* package.^23,79^ MLIPs for reactions R1 and R2 were trained with the energy selector with an energy threshold of 0.1 eV, while the similarity selector with a similarity threshold of 0.9995 using SOAP descriptor was applied for the reaction R3. MLIPs were evaluated using Atomic Simulation Environment (ASE) v3.23.0b1,^80^ and WTMetaD bias was calculated using the PLUMED plugin integrated with ASE.^80–82^ Hyperparameters of ACE, selectors and WTMetaD bias, used in training ACE MLIPs, are listed in Table S1. The MD-MLIP simulations in AL were performed at a constant temperature of 500 K in the case of Downhill AL and 300 K for WTMetaD-IB AL. Constant temperature MD was performed using Langevin dynamics with a friction coefficient of 0.02 in inverse ASE time units (
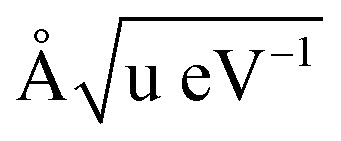
 where u is the atomic mass unit) and a timestep of 0.5 fs. Velocities were initialised using the Maxwell–Boltzmann distribution at the corresponding temperatures (Downhill AL at 500 K and WTMetaD AL at 300 K). More details on hyperparameters of MLIPs and WTMetaD in AL are provided in SI§S1.

Electronic structure calculations were performed using ORCA 5.0.3 ^83,84^ wrapped with autodE^85^*via mlp-train* package.^86^ The PBE0-D3BJ/def2-SVP^87–89^ method with Conductor-like Polarizable Continuum Model (CPCM)^90^ for water was selected as the ground-truth method for reaction R1. The IRC for the reaction R1 was obtained by directly using ORCA 5.0.3 at the same level of theory. The independent testing set was generated by US/AIMD for the reaction R1 with a reaction coordinate of *r*_Cl_ − *r*_F_, where *r*_Cl_ is the bond length between C and Cl atoms and *r*_F_ is the bond length between C and F atoms, with 16 windows, which were equally spaced in [−0.73, 1.85] Å with the force constant *k* = 15 eV Å^−2^, for 200 fs per window at 300 K in the NVT ensemble at the ground-truth level of theory. The PBE0-D3BJ/def2-SVP level of theory was used as the ground-truth method for R2. US/AIMD ran along *r*_1_ − *r*_2_ with 30 windows equally spaced in [−0.97, 1.03] Å with *k* = 20 eV Å^−2^, for 100 fs per window at 300 K at the same level of theory. The PBE-D3BJ/def2-TZVP^91,92^ method was used to label energies and forces during AL iterations, while the ωB97X-D3BJ/def2-TZVP^93^ was used to relabel all training data and as the ground-truth method for reaction R3.

### Free energy calculations

For R2, the US/MLIP-MD free energy profile was computed using *r*_1_ − *r*_2_ as the reaction coordinate, split into 30 equally-spaced windows with a force constant of 20 eV Å^−2^. The simulation ran for 40 ps at 365.6 K in every window and the first 10 ps in each window were used for equilibration and discarded from the analysis. Free energy was calculated by the weighted histogram analysis method (WHAM)^94^ as implemented in *mlp-train*. WTMetaD was performed along the same reaction coordinate, *r*_1_ − *r*_2_, as the US. The width of the deposited Gaussians was 0.07 Å with the initial height 
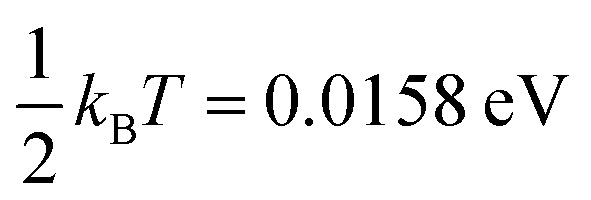
 at 365.6 K to be consistent with the experimental condition and the bias factor of 50. Gaussians were deposited every 100 fs. WTMetaD was run in 10 independent replicas for 500 ps each, starting from the same configuration but with different velocities initialised using the Maxwell–Boltzmann distribution. WTMetaD + IB was performed using the inherited bias from the 16th AL iteration as an initial bias. The parameters of the deposited Gaussians were the same, with the bias factor increased to 80. Ten independent WTMetaD + IB runs were performed with a simulation time of 250 ps each. The free energy profile was reconstructed by reweighting based on the final bias potential,^95^ using kernel density estimation as implemented in PLUMED with a bandwidth of 0.02 to compute the histograms.^81,82^

For R3, the free energy surface was investigated using WTMetaD/MLIP-MD. The simulation system comprised the substrate in 86 DCM molecules within a box with the size of 22 Å using periodic boundary conditions (PBC) to maintain the density of DCM (1.33 g cm^−3^). The box size was chosen to prevent the interaction of the substrate with its periodic copies. Before the WTMetaD simulation, the solvated system was optimised with the substrate fixed in its DFT-optimised geometry, followed by 5-ps MD dynamics in the NVT ensemble. The metadynamics parameters were taken from the study of Liu *et al.*,^71^ employing the coordination numbers (CNs)^96^ as CVs, namely the CN between C^1^ and O^LG^ and C^1^ and O^Nu^. The CN was defined as [1 − (*r*/*r*_0_)^6^]/[1 − (*r*/*r*_0_)^12^], with *r* representing the bond length of interest and *r*_0_ set at 2.5 Å. Additional constraints were imposed to maintain the bond lengths in a range of 1.35 Å to 5.0 Å to prevent sampling of the leaving and nucleophile too far or too close to the reaction centre. The WTMetaD simulations were conducted at 298 K, depositing a Gaussian potential with a height of 0.013 eV and width of 0.1 Å for both CNs with a bias factor of 50. The Gaussian was deposited every 25 fs. Three independent 300-ps simulations were executed, each initiating from the same configuration.

## Author contributions

VV, HZ, VJ, and FD conceptualised the study. VV implemented WTMetaD and WTMetaD-IB AL in the *mlp-train* package with the help of TJW. VV and HZ carried out the calculations with assistance from VJ and TJW. All authors participated in data analyses. VV, HZ, and VJ wrote the first draft. All authors contributed to the preparation of the manuscript. FD supervised the study.

## Conflicts of interest

There are no conflicts to declare.

## Supplementary Material

DD-005-D5DD00261C-s001

## Data Availability

The open-source *mlp-train* package is available at https://github.com/duartegroup/mlp-train and archived on Figshare (https://doi.org/10.6084/m9.figshare.25816864.v2). The training and testing datasets are available on Figshare (https://doi.org/10.6084/m9.figshare.28631591), along with detailed instructions and ready-to-run Python scripts for reproducibility. The dataset consists of three folders (R1–R3), each corresponding to one reaction. Each folder contains Input geometries (.xyz files), Python scripts for training, and ground-truth electronic energy data (energy and forces). For R1 and R2, AL training was performed using both downhill and WTMetaD-IB sampling, whereas R3 was trained exclusively *via* WTMetaD-IB-based AL; the relevant scripts are located within subfolders named after the sampling methods (*e.g.*, al_downhill). Additionally, each folder also includes MLIP-predicted energies and forces (*npz format). For R2, free energy calculations were performed by three enhanced sampling techniques (umbrella sampling, WTMetaD and WTMetaD-IB). Input files and configurations for each of them are organised into their own subfolders. For reaction R3, the initial and final configurations from the trajectories, used to investigate the solvent-separated ion pair (Fig. 6c in the main text), are included. Supplementary information (SI): detailed settings for the hyperparameters employed in MLIP training, along with a comparison of sampling methods used in active learning strategies. It also provides detailed analyses of the sampled PES and convergence of the free energy calculations. See DOI: https://doi.org/10.1039/d5dd00261c.
