# Supplementary material for: Active learning meets metadynamics: automated workflow for reactive machine learning interatomic potentials
Source: Digit Discov. 2025 Oct 30;5(1):108–22. doi: 10.1039/d5dd00261c (PMC12642453; doi:10.1039/d5dd00261c)
Supplement: DD-005-D5DD00261C-s001 [file DD-005-D5DD00261C-s001.pdf]

Supplementary Information (SI) for Digital Discovery.
This journal is © The Royal Society of Chemistry 2025

Supporting Information

Active learning meets metadynamics: Automated workflow for reactive machine learning interatomic potentials

Valdas Vitartas, Hanwen Zhang, Veronika Juraskova, Tristan Johnston-Wood and Fernanda Duarte

Contents

S1 Hyperparameters **S2**

S2 R1: S_N2 reaction between fluoride and chloromethane **S4**

S2.1 Training protocol and accuracy evaluation S4

S2.2 WTMetaD AL S8

S3 R2: 2,2-dimethylisoindene methyl shift **S11**

S3.1 Training protocol and accuracy evaluation S11

S3.2 Free energy calculations S14

S4 R3: Glycosylation reaction in explicit DCM **S17**

S4.1 Training protocol and accuracy evaluation S17

S4.2 Free energy calculations S24

S4.2.1 PBE-D3BJ/def-TZVP level of theory S24

S4.2.2 ωB97X-D3BJ/def2-TZVP level of theory S26

S4.3 Accuracy of MLIP with chloride counterion S29

S1 Hyperparameters

Table S1 presents the hyperparameter settings used in training machine learning potentials (MLIP), which are also the default values in the *mlp-train* package [1], unless otherwise specified. These hyperparameters are divided into three parts: ACE MLIPs, active learning (AL) selectors including the Smooth Overlap of Atomic Positions (SOAP) descriptor applied in the *similarity* selector, and well-tempered metadynamics (WTMetaD). Some of these hyperparameters have been validated in our previous works [1, 2].

For the ACE MLIPs, two cutoff functions are applied. The first one controls the many-body potentials and consists of an inner and outer cutoff radius. In the *mlp-train* package, the inner cutoff radius is determined based on the minimum pairwise distance observed in the training data. The outer cutoff radius r_{mb} in Table S1 sets the maximum distance at which the many-body potentials are considered. The second cutoff function is applied to the pair potential with a default setting of 5 Å, aiming to capture interactions of longer and very short ranges in the system. For the reaction **R3**, this hyperparameter is set to 5.5 Å to encompass longer two-body interactions. The weight ratio between energy and forces is set to 20.0 in the loss function. Additionally, the loss function incorporates L_2 regularization with a penalty weight of 0.1.

Table S1: Hyperparameter setting for ACE potential, selectors and WTMetaD in training MLIPs. (* the polynomial degree for four- and five-body potentials do not include H; † adjustment for the reaction **R3**, § bias factor for **R1**, **R2** and **R3**, respectively)

Type	Parameter	Description	Value
ACE	ν	Maximum correlation order	4
	$D_{\nu}^{\max}$	Maximum polynomial degrees for corresponding body potential	20, 16, 16, 12 *
	r_{mb}	Outer cutoff radius for many-body potentials	4.0 Å
	r_{pair}	Cutoff radius for the pair potential	5.0 (5.5 †) Å
energy selector	E_T	Selection threshold	0.1 eV
similarity selector	k_T	Selection threshold	0.9995
SOAP descriptor	$\sigma_{\text{at}}^{\text{SOAP}}$	Spread of the Gaussian added to atomic density	1.0 Å
	$n_{\max}$, $l_{\max}$	The maximum number and degree for the radial (n) and angular (l) basis	6
	r_{cut}	Cutoff distance for local region	5.0 Å
WTMetaD	σ	width, standard deviation of the placed Gaussian	0.05
	τ_{G}	pace, interval at which a new Gaussian is placed	10 fs
	ω	height, initial height of placed Gaussians	$5k_{\text{B}}$T
	γ	bias factor, describes how quickly Gaussians shrink	100, 90, 70 §

S2 R1: S_N2 reaction between fluoride and chloromethane

S2.1 Training protocol and accuracy evaluation

For reaction **R1**, the AL coupled to MLIP molecular dynamics (MLIP-MD) downhill is initiated from the density functional theory (DFT)-optimised transition state (TS) and propagating downhill to reactant state (RS) and product state (PS). As the trajectory propagates from a high to a low energy region, a relatively short MLIP-MD simulation time, approximately several hundred femtoseconds (fs), is sufficient to sample the relevant potential energy surface (PES), reaching both RS and PS without bias.

The MLIP was trained using the inherited bias well-tempered metadynamics (WTMetaD-IB) AL method with collective variable (CV) corresponding to $r_{\mathrm{Cl}} - r_{\mathrm{F}}$, where r_{Cl} represents the bond length between atoms C and Cl and r_{F} bond length between atom C and atom F. Since the iterative WTMetaD-IB AL process starts from RS, it is crucial to introduce a sufficiently large bias to overcome the energy barrier so that it can explore both the TS and RS.

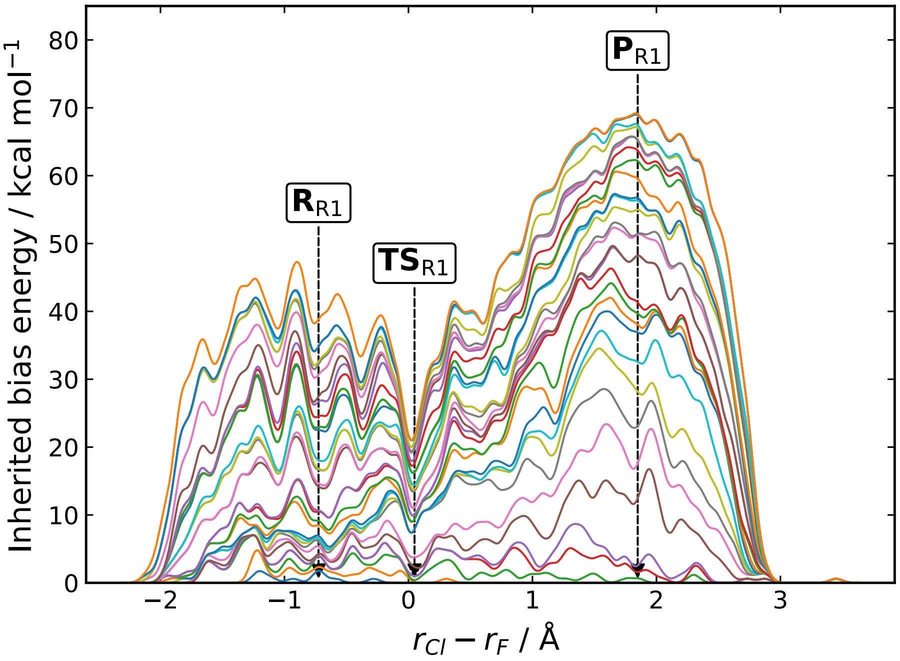

Figure S1: Inherited bias surfaces generated during 33 WTMetaD-IB AL iterations for the reaction **R1**. Five parallel processes were used during AL, which means each surface is an average of five bias surfaces.

Fig. S1 illustrates the inherited bias surface generated over 33 AL iterations, which increases along the AL iterations, particularly in the RS and PS regions. This bias, along with the updated starting points in MLIP-MD, ensures adequate sampling even within the fs to ps MLIP-MD simulations. This is further supported by the trajectories of deposited Gaussians (shown in Fig. S2), confirming the successful sampling of the RS, TS and PS along the reaction pathway in the generation of training data. Fig. 2 and 3b in the main manuscript depict the locations and distribution of the

training data, respectively, further validating the comprehensive coverage of the PES in WTMetaD-IB.

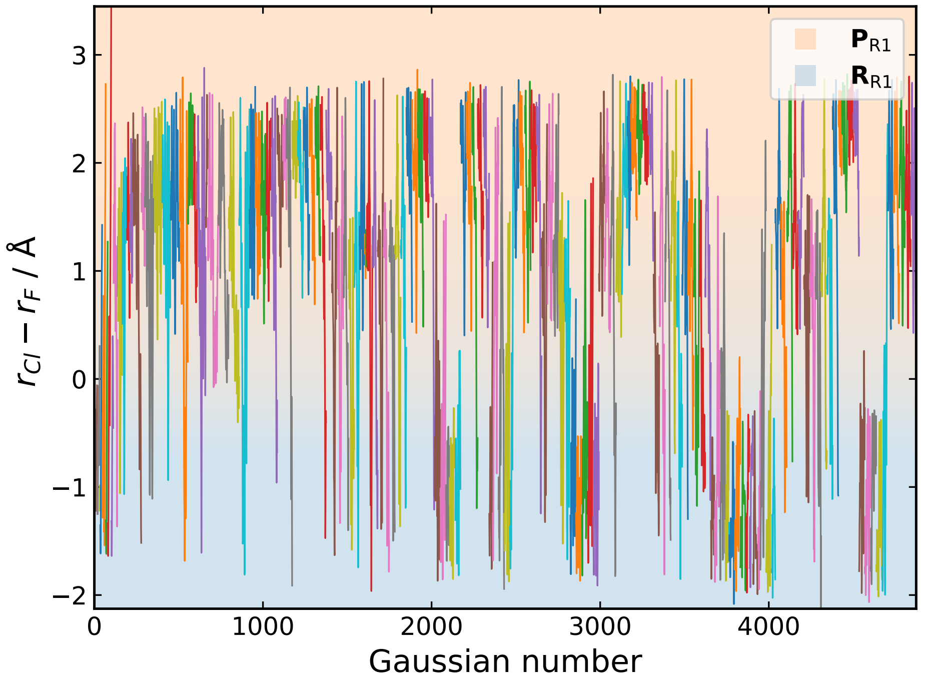

Figure S2: Sampling of the relevant regions of the PES, described by $r_{\mathrm{Cl}} - r_{\mathrm{F}}$, with an increasing number of deposited Gaussians during WTMetaD-IB AL for reaction **R1**. Each colour denotes a separate MD process during which the Gaussians were deposited.

Two datasets, IRC and umbrella sampling (US) driven by *ab initio* molecular dynamics (AIMD) (US/AIMD), were used to validate the performance of the resulting MLIPs. The IRC dataset represents the minimum energy pathway (MEP) in mass-weighted coordinates. However, MD simulations reveal that trajectories from RS to PS do not strictly follow the MEP. This deviation is illustrated in Fig. S3, which compares the Cl–C–F angle across IRC and dynamic sampling configurations, including downhill and WTMetaD-IB AL training data, as well as US testing data. While the IRC maintains a constant angle of approximately 180°, dynamic sampling configurations exhibit deviations. Notably, the two training data sets present configurations with greater deviations from 180° compared to the US testing data.

In addition to evaluating the accuracy of MLIPs with downhill and WTMetaD-IB sampling, their stability has been assessed through 100-ps NVE MLIP-MD, as illustrated in Fig. S4. The potential energy fluctuations range from -0.2 eV to 0.2 eV, while the total energy remains constant with minimal fluctuations throughout the 100 ps simulation for both MLIPs, thereby confirming their stability.

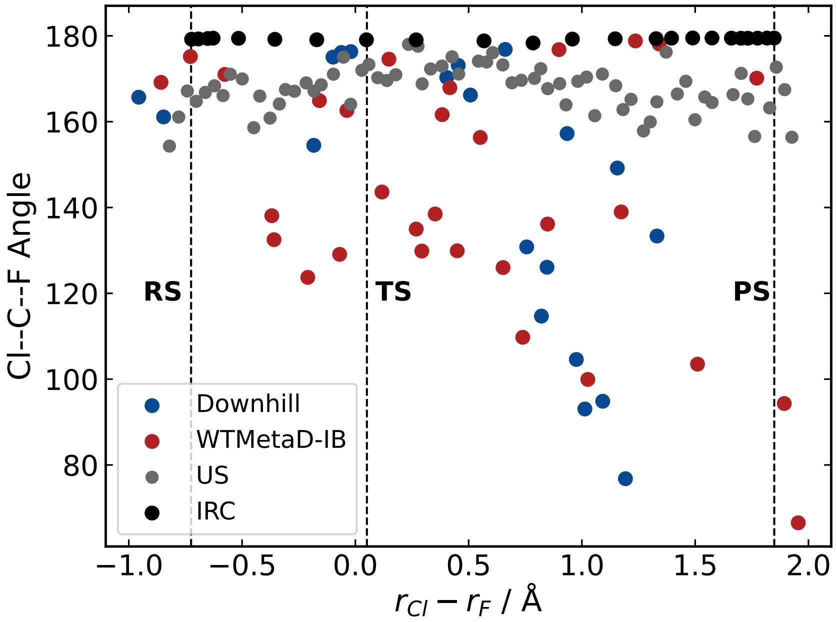

Figure S3: Angles of Cl–C–F for configurations in the downhill AL (blue) and WTMetaD-IB (red) training data, US testing data (grey) and IRC (black) along $r_{\mathrm{Cl}} - r_{\mathrm{F}}$. The positions of RS, TS and PS are highlighted by the dashed lines.

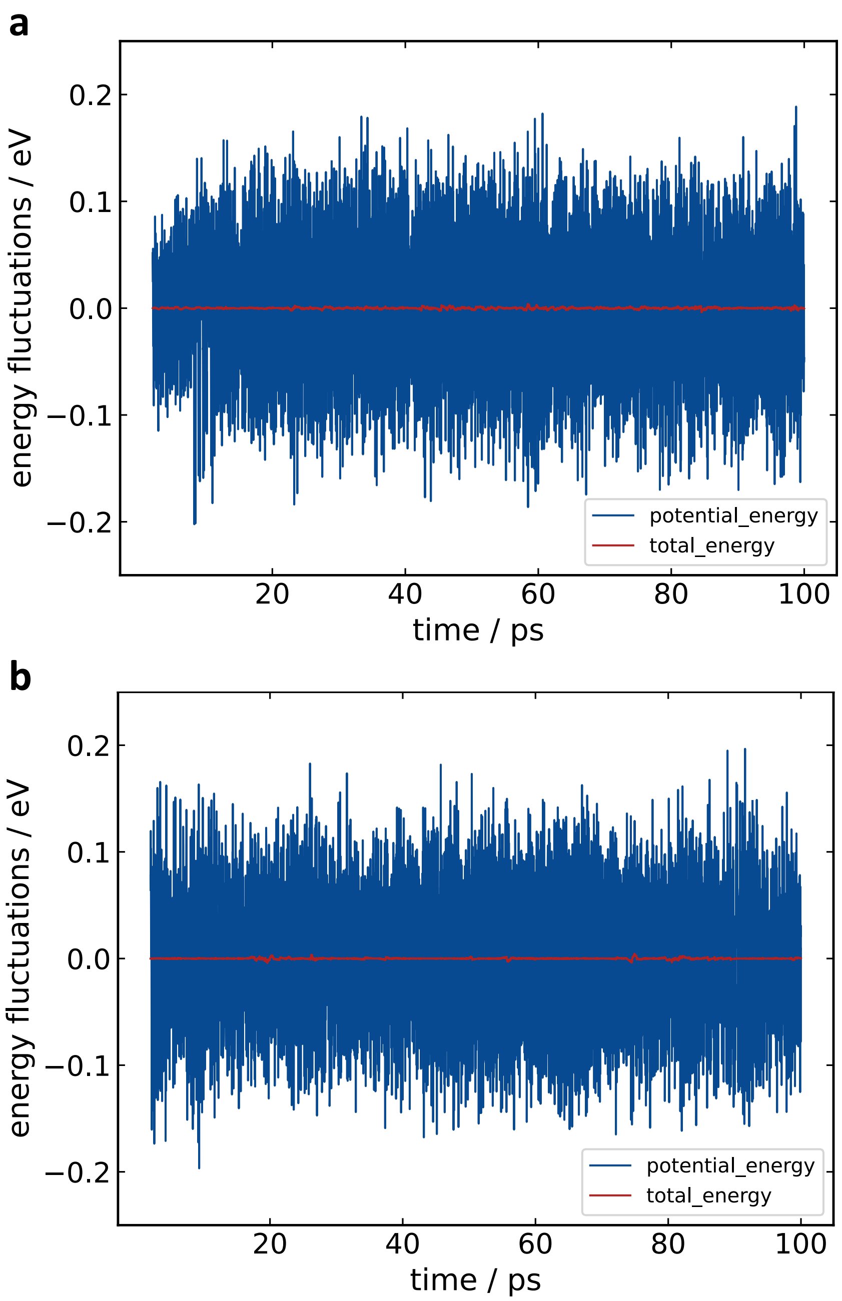

Figure S4: Energy fluctuations of the MLIP trained by (a) downhill and (b) WTMetaD-IB sampling for the **R1** during 100 ps NVE simulation

S2.2 WTMetaD AL

To evaluate the proposed WTMetaD-IB AL in comparison with standard WTMetaD AL—where no bias is inherited and no starting points are updated—another MLIP for reaction **R1** was trained using the WTMetaD AL method. Besides the key differences, including the absence of inherited bias and the utilization of reactants as fixed initial points in MLIP-MD, the maximum simulation time for MLIP-MD in AL was extended from 1 ps to 1.5 ps to ensure adequate sampling of the phase space region.

Table S2: Training details and accuracy tested on the US data for MLIP trained with downhill, WTMetaD-IB and WTMetaD (46 AL iterations) sampling methods for **R1**, with CPU Architecture of Intel(R) Xeon(R) Gold 5218R CPU @ 2.10 Hz

Sampling Method	# Iterations	# Configs	Training time / CPUhs	Energies MAE / meV atom^{-1}	Forces MAE /meV Å^{-1}
Downhill	21	45	240	2.14	115.74
WTMetaD-IB	33	78	250	1.83	93.29
WTMetaD	46	130	462	2.22	119.42

Table S2 lists the AL details and accuracy metrics for MLIPs trained by downhill, WTMetaD-IB and WTMetaD AL. The WTMetaD AL method is the most computationally intensive, requiring 462 CPU hours, nearly twice the time of both the downhill and WTMetaD-IB methods. This method also concluded after 46 iterations, generating 130 training configurations, which is approximately double the training data of WTMetaD-IB AL and triple that of downhill AL. Despite the increased training data and computational resources, WTMetaD AL does not yield a more accurate MLIP. Validation of these MLIPs was conducted using the same testing dataset, generated via short umbrella sampling driven by the ground-truth method (US/AIMD), which is CPCM(water)-PBE0-D3BJ/def2-SVP. The MLIP trained with WTMetaD AL exhibited the poorest accuracy, with an energy error of 2.22 meV atom^{-1} and a force error of 119.42 meV Å^{-1}.

To establish the origins of the high error in the MLIP trained by WTMetaD AL, the accuracy of energies and forces along the reaction coordinate were illustrated in Fig. S5. The largest errors are observed near the RS, with an energy MAE of 6.51 meV atom^{-1} and a force MAE of 193.61 meV Å^{-1}, and near the PS, with an energy MAE of 5.05 meV atom^{-1} and a force MAE of 199.40 meV Å^{-1}. Fig. S6 compares the training data distributions of MLIPs trained by WTMetaD and WTMetaD-IB AL. Both datasets cover critical regions of the PES. Therefore, the high error observed in RS and PS for the MLIP trained by WTMetaD is not due to insufficient sampling. Surprisingly, WTMetaD AL collects a larger number of configurations near the PS region compared to WTMetaD-IB AL. However, increased sampling in the PS does not result in higher accuracy in the region. This is attributed to the sampling of highly distorted geometries (illustrated in S6 right panel) and fewer stable PS configurations during WTMetaD AL. These

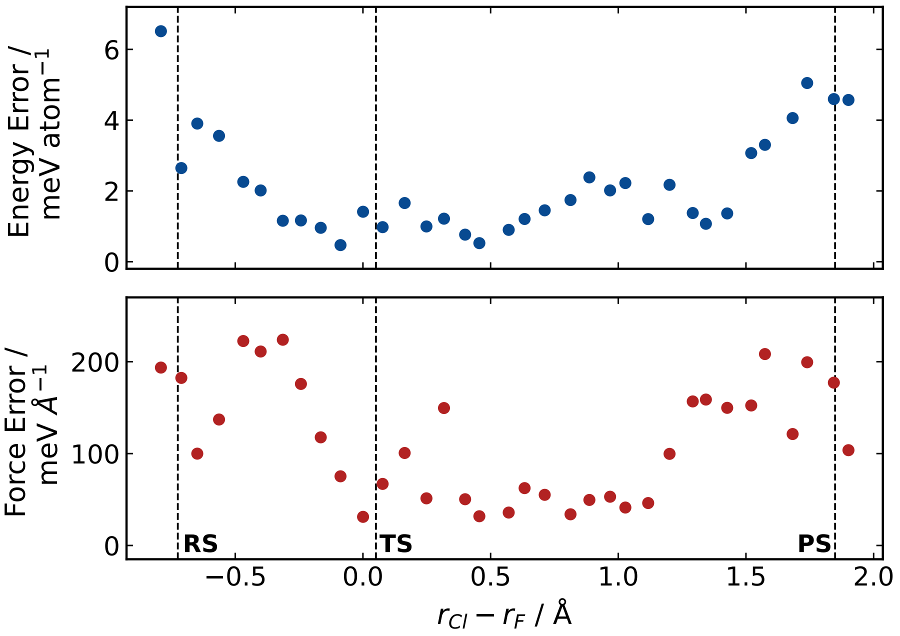

Figure S5: Energy errors (upper panel) and force errors (bottom panel) for the independent testing dataset generated by US/AIMD along the CV of $r_{\mathrm{Cl}} - r_{\mathrm{F}}$, which was used in WTMetaD AL for the MLIP of reaction **R1** The positions of RS, TS and PS are highlighted by the dashed lines.

highly distorted configurations are not representative of those generated in dynamics simulations, such as the US/AIMD testing dataset. This suggests that a more detailed exploration, starting from relevant areas, may be necessary in an enhanced-sampling integrated AL strategy. Due to computational intensity and inaccuracy, WTMetaD AL will not be used for training MLIPs in the following reactions.

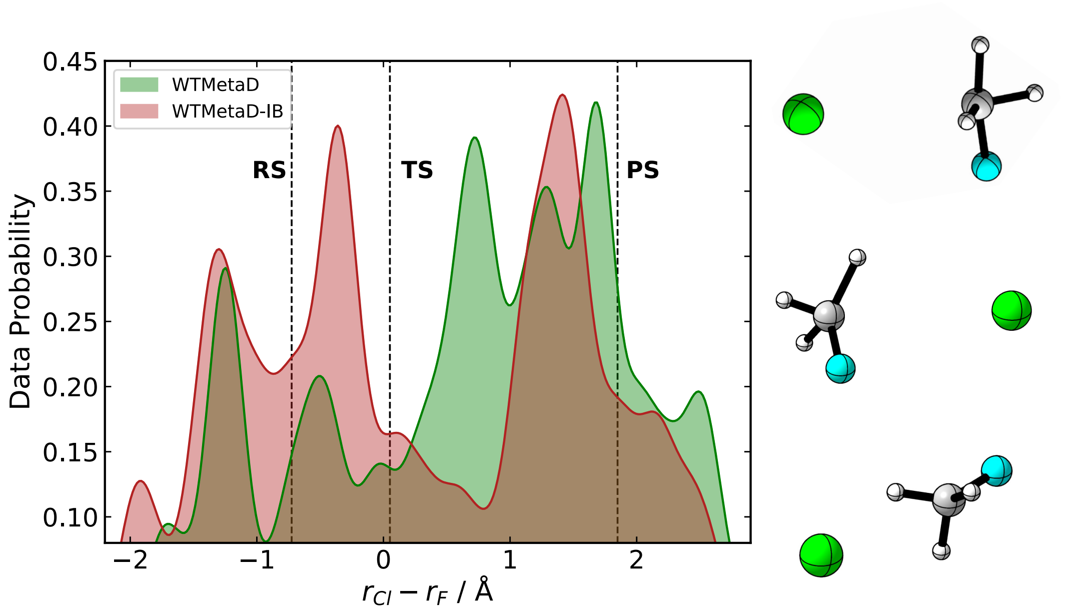

Figure S6: Data distribution of training data for the two MLIPs trained by WTMetaD and WTMetaD-IB AL. Three representative product geometries sampled during WTMetaD AL are illustrated in the right panel

S3 R2: 2,2-dimethylisoindene methyl shift

S3.1 Training protocol and accuracy evaluation

Similar to Reaction **R1**, MLIPs for reaction **R2** were trained using both downhill and WTMetaD-IB AL methods, with the bond length difference, $r_1 - r_2$ as the CV in WTMetaD-IB AL. Fig. S7 illustrates the evolution of inherited bias during WTMetaD-IB AL, showing a consistent increase of the updated bias throughout the AL process. As more Gaussians are deposited, above 4000, enough data points were gathered to cover different regions of the PES (Fig. S8). This is further corroborated by Fig. 4a, which shows the distribution of training data points generated by WTMetaD-IB AL.

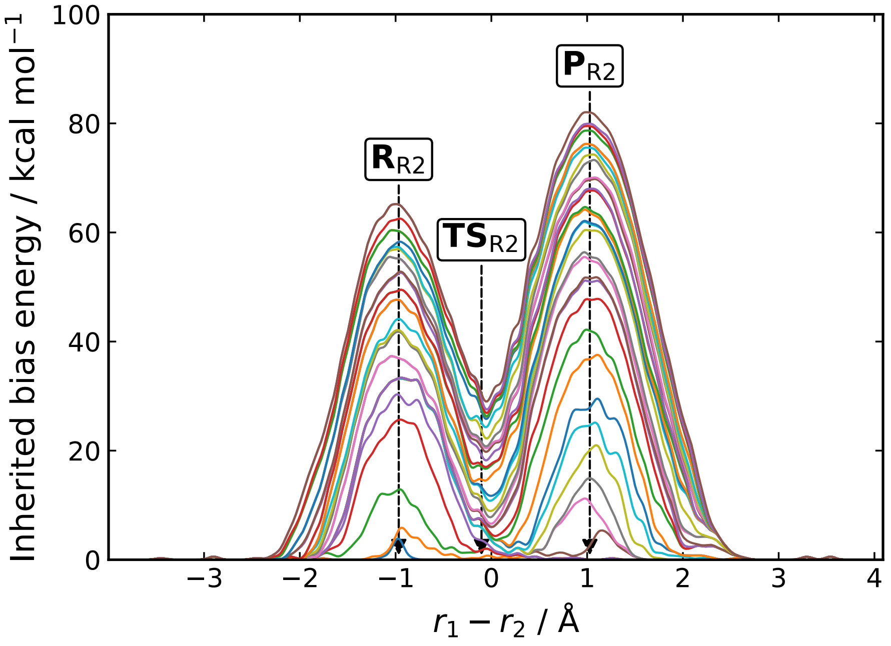

Figure S7: Inherited bias generated during 37 WTMeatD-IB AL iterations for the reaction **R2**. Each surface represents the average of five bias surfaces.

Table S3 summarises the accuracy metrics for MLIPs trained using downhill and WTMetaD-IB AL strategies on test data generated by US/AIMD. The WTMetaD-IB AL strategy required 14 additional iterations, adding 61 training configurations and consuming 55 more CPU hours than downhill MD. Despite this, MLIPs trained with WTMetaD-IB AL achieved higher accuracy, with energy errors of 1.49 meV atom^{-1} and force errors of 161.76 meV Å^{-1}, compared to the 1.76 meV atom^{-1} and 175.10 meV Å^{-1} for the downhill.

Fig. S9 shows the energy and force errors on the US/AIMD testing data, along with the training data distribution in the CV space of $r_1 - r_2$ for both downhill and WTMetaD-IB AL methods. Two sampling gaps are observed in the CV space for the downhill AL method: one between RS and TS, and another between TS and PS. These gaps correlate with higher energy errors, exceeding the

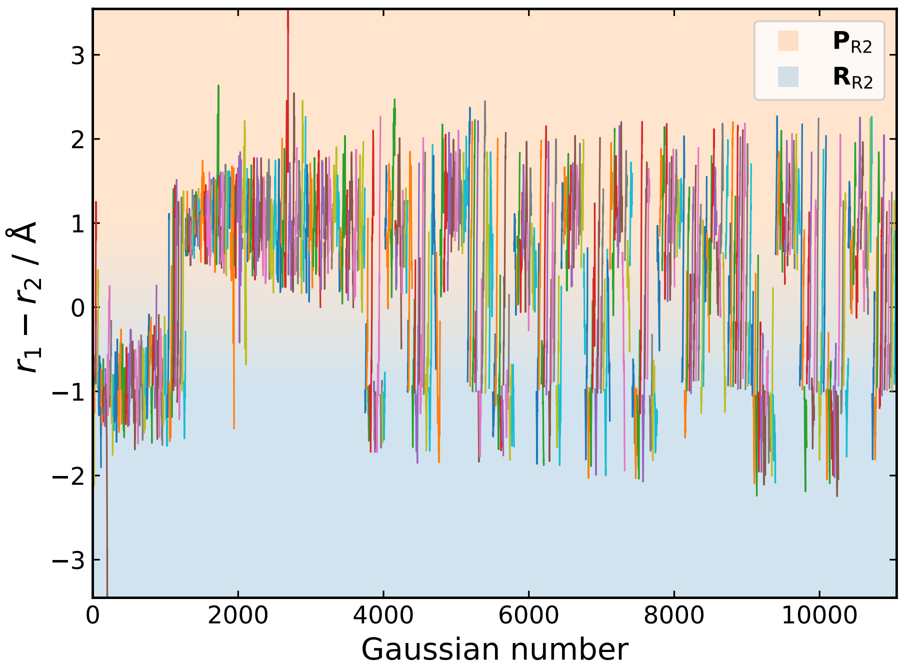

Figure S8: Sampling of the relevant regions of the PES, described by $r_1 - r_2$, with an increasing number of deposited Gaussians during WTMetaD-IB AL for reaction **R2**. Each colour denotes a separate MD process during which the Gaussians were deposited.

Table S3: Training details and accuracy of MLIPs trained with downhill and WTMetaD-IB AL for **R2** tested on the US/AIMD data, using an Intel(R) Xeon(R) Gold 6126 CPU @ 2.60 Hz

Sampling Method	# Iterations	# Configs	Training time / CPUhs	Energies MAE / meV atom^{-1}	Forces MAE /meV Å^{-1}
Downhill	23	131	200	1.76	175.10
WTMetaD-IB	37	192	255	1.49	161.76

chemical accuracy threshold of 1.87 meV atom^{-1}. Conversely, no sampling gaps are detected for the WTMetaD-IB AL method, and its MLIP achieves energy prediction accuracy close to chemical accuracy, except for two outliers near the TS region (Fig. S9). The enhanced performance of the WTMetaD-IB MLIP is attributed to more uniform sampling in the CV space.

To summarize the performance of MLIPs on reactions **R1** and **R2**, Table S4 reports the MAEs in energies and forces for models trained with downhill sampling and WTMetaD-IB AL. For both **R1** and **R2**, the MLIPs trained using WTMetaD-IB show better overall performance on the full umbrella sampling (US)/AIMD test set (*Overall* in Table S4, energies 1.83 vs. 2.14 meV atom^{-1} for **R1** and 1.49 vs. 1.76 meV atom^{-1} for **R2**) However, when comparing performance in the TS region, the MLIPs trained with downhill sampling demonstrate superior performance in the

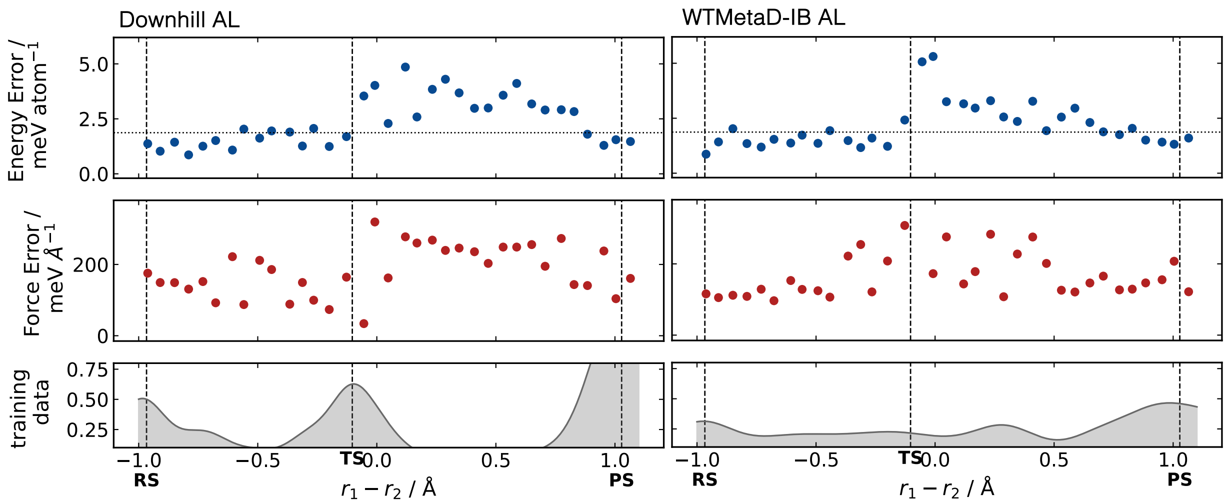

Figure S9: Energy error (upper panel) and force error (middle panel) for the test set generated by US/AIMD, and the training data distribution (bottom panel) along the collective variable (CV) $r_1 - r_2$ for MLIPs trained using downhill (right panel) and WTMetaD-IB (left-panel) AL. Chemical accuracy (1.87 meV atom^{-1}) is represented by the dotted line in the upper panel. The positions of RS, TS and PS are indicated by dashed lines.

US/AIMD test set for both **R1** and **R2**, with MAEs of 1.71 vs. 3.55 meV atom^{-1} for **R1** and 3.08 vs. 4.27 meV atom^{-1} for **R2**. Such a performance would be in line with the fact that downhill sampling explicitly includes information of this region in the training. Still, the fact that the MLIP trained with WTMetaD-IB AL shows better performance on the IRC test dataset (2.12 vs 2.66 meV atom^{-1} in overall performance and 2.37 vs 2.63 meV atom^{-1} at the TS point), suggests that this MLIP provides competitive performance.

Table S4: Mean absolute errors (MAE) for MLIPs trained by downhill and WTMetaD AL strategy for reaction **R1** and **R2**, considering overall performance on the full umbrella sampling (US)/AIMD test set and performance specifically within the TS region of the US test set. Values in parentheses correspond to MAEs on the whole IRC test set for **R1**; and the error at the true TS point. MAE energies are given in meV atom^{-1} and forces in meV Å^{-1}.

	**Reaction R1**				**Reaction R2**			
	Overall		TS region		Overall		TS region	
	Energy	Force	Energy	Force	Energy	Force	Energy	Force
Downhill AL	2.14(2.66)	115.74	1.71(2.63)	74.99	1.76	175.10	3.08	172.54
WTMetaD AL	1.83(2.12)	93.29	3.55(2.37)	185.77	1.49	161.76	4.27	290.24

S3.2 Free energy calculations

The free energy surface (FES) for **R2** was computed using different enhanced methods, including US, WTMetaD, and WTMetaD with an inherited bias (WTMetaD+IB). In the latter method, the bias generated during the WTMetaD-IB AL process served as the initial bias for WTMetaD simulations. Each enhanced sampling method was performed ten times to establish confidence intervals and evaluate the convergence of the FES. The following sections provide a detailed analysis of these methodologies.

US

US/MLIP-MD simulations were conducted using a reaction coordinate of $r_1 - r_2$, which was divided into 30 windows ranging from -0.97 to 1.03. For each window, MLIP-MD simulations were performed in the NVT ensemble at 365.6 K for 40 ps, resulting in a cumulative simulation time of 1.2 ns. This duration significantly exceeds the 1 ps used in the exploration step of AL. The obtained MLIP exhibited excellent stability across all ten runs.

Fig. S10 presents the histograms from one US/MLIP-MD simulation, demonstrating sampling coverage from RS to PS. The confidence interval was derived from ten repetitions of the US simulations, applying the central limit theorem (CLT) to compute the standard deviation of the mean free energy. Fig. 4b illustrates the 95% confidence interval as a shaded region, which is 0.05 kcal mol^{-1}.

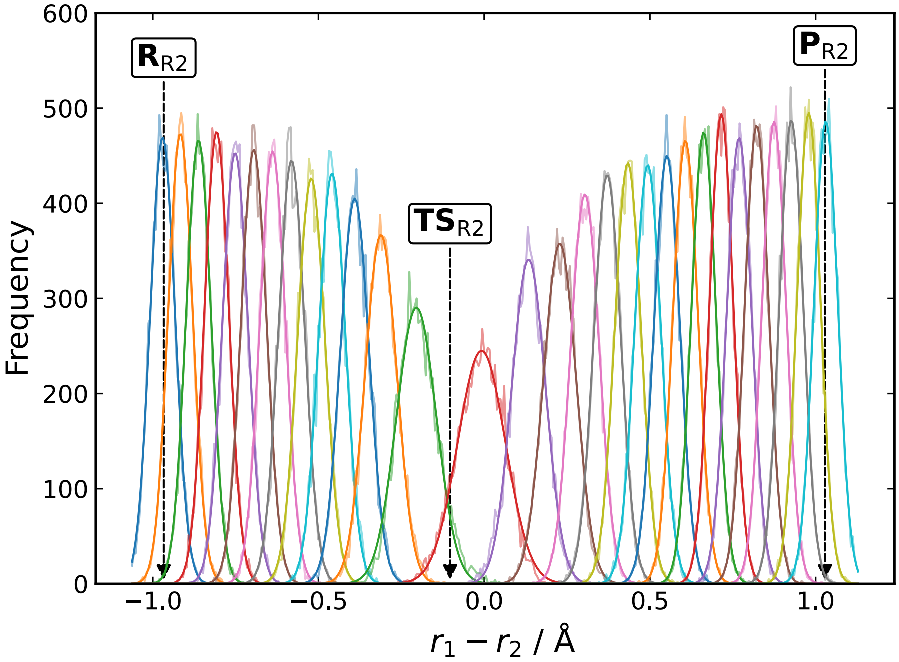

Figure S10: Histogram along the reaction coordinate for 30 umbrella window in **R2**. The positions of RS, TS and PS are highlighted by dashed lines.

WTMetaD

WTMetaD/MLIP-MD simulations were conducted using the CV of $r_1 - r_2$ in the NVT ensemble at 365.6 K for 500 ps. Among ten independent WTMetaD runs, nine remained stable, while one resulted in unphysical configurations. The free energy surface was derived by correlating the deposited bias with the free energy [3]. The standard deviation of the activation free energy, calculated with a 95% confidence interval using the CLT, was 0.32 kcal/mol.

The left panel of Fig. S11 depicts the standard deviation of the FES as a function of block size, derived from block averaging analysis [4] conducted on the final 300 ps of the trajectory from a stable run. In this run, the standard deviation plateaus at a block size of approximately 1500, with a value of 0.09 kcal, indicating the convergence of this WTMetaD simulation.

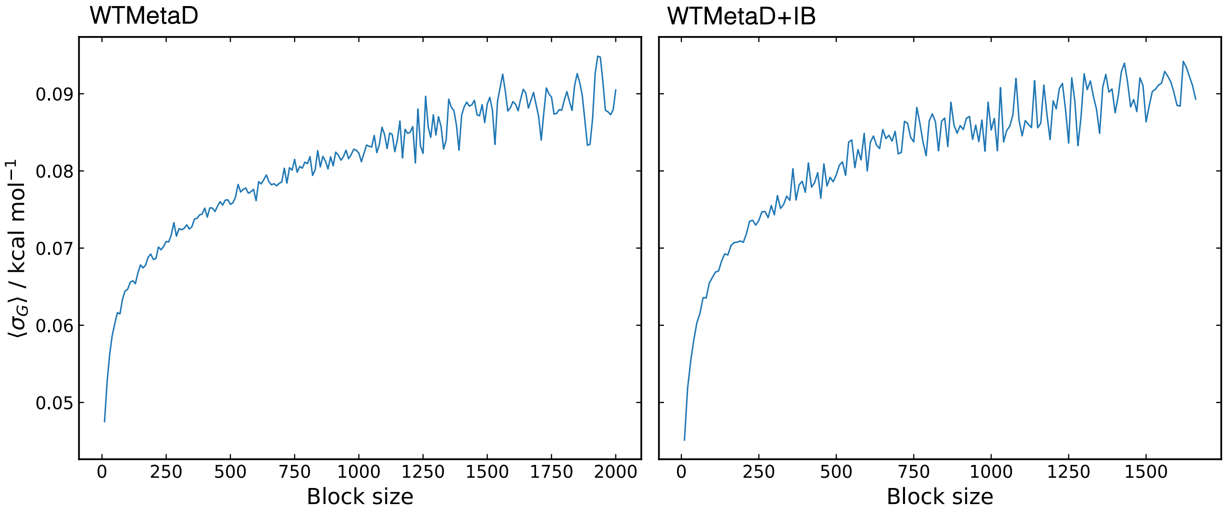

Figure S11: Block averaging analysis on the standard deviation of WTMetaD/MLIP-MD simulation (left panel) on the final 300 ps trajectory and WTMetaD+IB/MLIP-MD simulation (right panel) on the 250 ps trajectory for **R2**.

WTMetaD+IB

The inherited bias generated during the WTMetaD-IB AL process can be used as an initial bias in WTMetaD simulations, referred to as WTMetaD+IB simulations in this study. In reaction **R2**, the inherited bias from the 16th AL iteration was applied as a starting bias. Similar to the WTMetaD simulation without an initial bias, 9 out of 10 runs remained stable. These stable runs were used to calculate the 95% confidence interval, yielding standard deviations in activation free energy of 0.43 kcal mol^{-1}.

A block averaging analysis of the standard deviation for the entire trajectory from one of the stable runs is presented in the right panel of Fig. S11. The error stabilises around a block size of 1500, with a value of 0.09 kcal mol^{-1}, indicating the convergence of the simulation with half the simulation time compared to the WTMetaD simulation.

Fig. S12 displays the trajectories of reaction **R2** during the WTMetaD (left panel) and WT-MetaD+IB (right panel) simulation. The WTMetaD+IB simulation shows that the trajectory spans from the RS to the PS at the onset, whereas, in the WTMetaD simulation, the trajectory begins to visit the PS after 50 ps. This explains why WTMetaD+IB achieves more efficient convergence than WTMetaD simulation.

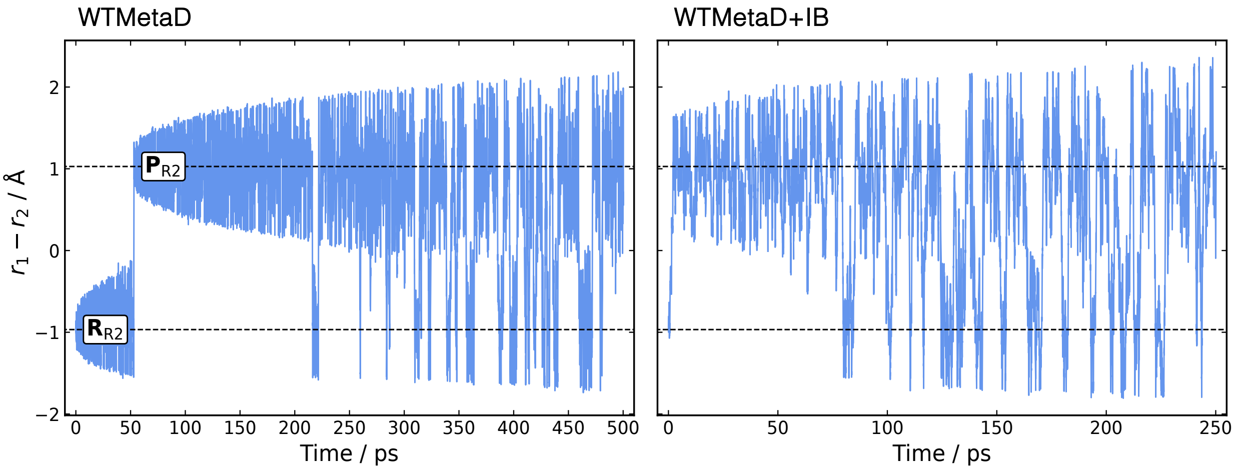

Figure S12: Trajectory of the reaction **R2** during WTMetaD and WTMetaD+IB simulations. RS and PS are marked by dotted lines.

S4 R3: Glycosylation reaction in explicit DCM

S4.1 Training protocol and accuracy evaluation

For reaction **R3**, the dependence of the mechanism on the solvent provides an ideal test case for our WTMetaD+IB AL approach, as it ensures that the data collected for training reflect the different pathways available **R3**. To achieve a data-efficient and stable MLIP, we adopted the training strategy from our recent work [2], where three independent training data were generated and then combined, each capturing different aspects of the reaction under study. Subset 1 included gas-phase substrate configurations to describe the intrinsic reactivity of the system. Subset 2, comprising the reactive substrate and 44 DCM molecules, accounts for reactivity and solvent-solute interactions. Subsets 1 and 2 were created using WTMetaD AL with the CV of bond length difference between $r_{\mathrm{C^1-O^{LG}}}$ and $r_{\mathrm{C^1-O^{Nu}}}$. Finally, subset 3, including 28 DCM molecules, provides information on solvent-solvent interactions.

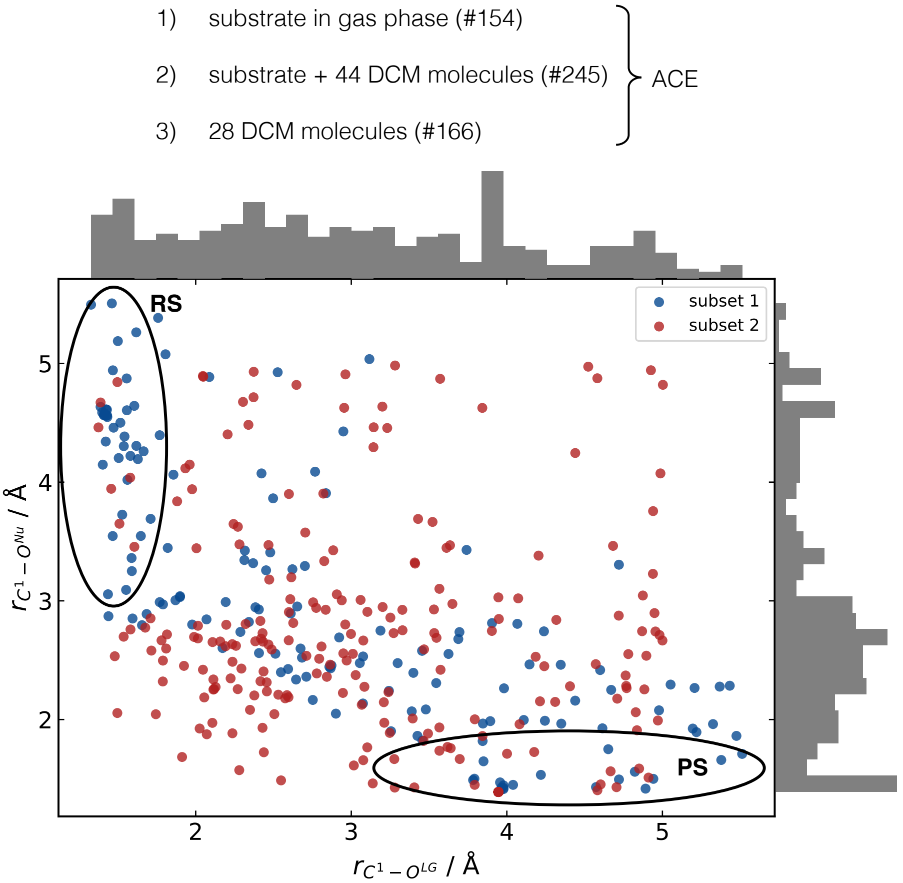

Figure S13: Distribution of training points of **R3** generated by WTMetaD+IB AL in the gas phase (represented by blue dots) and in explicit DCM (illustrated by red dots). The Histograms depict the distribution of bond lengths of $r_{\mathrm{C^1-O^{LG}}}$ (top) and $r_{\mathrm{C^1-O^{Nu}}}$ (right)

The *similarity* selector [2] was used for data collection during AL. For subset 1, training started from the RS in the gas phase; after 27 AL iterations, subset 1 accumulated 154 configurations.

Training for subset 2 began with the RS and 44 randomly located DCM molecules in a cubic box with a length of 18.5 Å. Before the WTMetaD-IB AL, six AL iterations were performed with the substrate fixed to capture rare solvent-solute interactions caused by the random displacement of DCM molecules, followed by 49 AL iterations of WTMetaD-IB. This resulted in overall 245 configurations. For subset 3, training began with 28 randomly located DCM molecules in a box of size length 14.5 Å. Given the absence of chemical reactions in subset 3, no bias was applied during AL. After 25 AL iterations, subset 3 yielded 166 configurations. Periodic boundary conditions (PBC) were applied to generated subsets 2 and 3 in AL. To minimise the computational demands of generating DFT training labels, cluster configurations were used by setting the box size length to 100 Å for both subsets. During AL iteration, PBE-D3BJ/def2-TZVP was used to label the configurations with energies and forces. The ACE MLIP was trained using data from subsets 1, 2, and 3 with a total of 565 configurations.

Fig. S13 displays the distribution of configurations in subsets 1 (blue dots) and 2 (red dots), demonstrating they cover both RS and PS regions as well as regions in between. Configurations with bond lengths $r_{\mathrm{C^1-O^{LG}}}$ and $r_{\mathrm{C^1-O^{Nu}}}$ exceeding 4 Å, indicating a loose contact ion pair were only obtained in subset 2, suggesting that explicit DCM solvent plays a role in stabilising this state. Since the training data covered the relevant regions in the PES, the resulting MLIP could accurately describe **R3** in explicit DCM solvent.

For validation, a system consisting of the substrate and 56 DCM molecules within a 19.6 Å box, depicted in the lower left corner of Fig. S14, was selected. This system differs from the training system, thus preventing data leakage, as it contains 56 DCM molecules and is computationally affordable for QM calculations. The configurations in test set trajectories were prepared by uphill dynamics as follows. Firstly, the solvent was minimised with a fixed substrate, followed by 1 ps of equilibration with step size of 0.5 fs in the NVT ensemble. Afterwards, four independent MLIP-MD uphill trajectories were initiated, two from the RS and two from the PS, using the NVT ensemble at 300 K with a 0.5 fs time step. For each two trajectories initialised from RS or PS, harmonic potentials were applied in all simulations to overcome energy barriers in respective directions, with force constants of 1.5 eV Å^{-2} and 2 eV Å^{-2}, and propagated for 1.5 ps and 1.0 ps, respectively, resulting in 1004 testing data points.

Fig. S14 depicts the locations of 1,004 testing data points categorised into four distinct classes: The region where $r_{\mathrm{C^1-O^{LG}}}$ is less than 2 Å is designated as the RS (purple). Similarly, the region where $r_{\mathrm{C^1-O^{Nu}}}$ is less than 2 Å is labelled as the PS (blue). Points not falling into these two categories are classified as intermediates. Among these intermediates, points where $r_{\mathrm{C^1-O^{LG}}}$ is less than $r_{\mathrm{C^1-O^{Nu}}}$ are closer to the RS and are designated as Inter1 (green). Conversely, points closer to the PS, where $r_{\mathrm{C^1-O^{Nu}}}$ is smaller, are classified as Inter2 (yellow). Fig. S15 employs the same classification and colour scheme to represent the SOAP [5] descriptor of this system while highlighting MLIP energy errors. The resulting potential with good accuracy of MAD of energy and forces are 0.57 meV atom^{-1} and 37.25 meV Å^{-1}, respectively. The largest energy errors are in the PS region (with 207

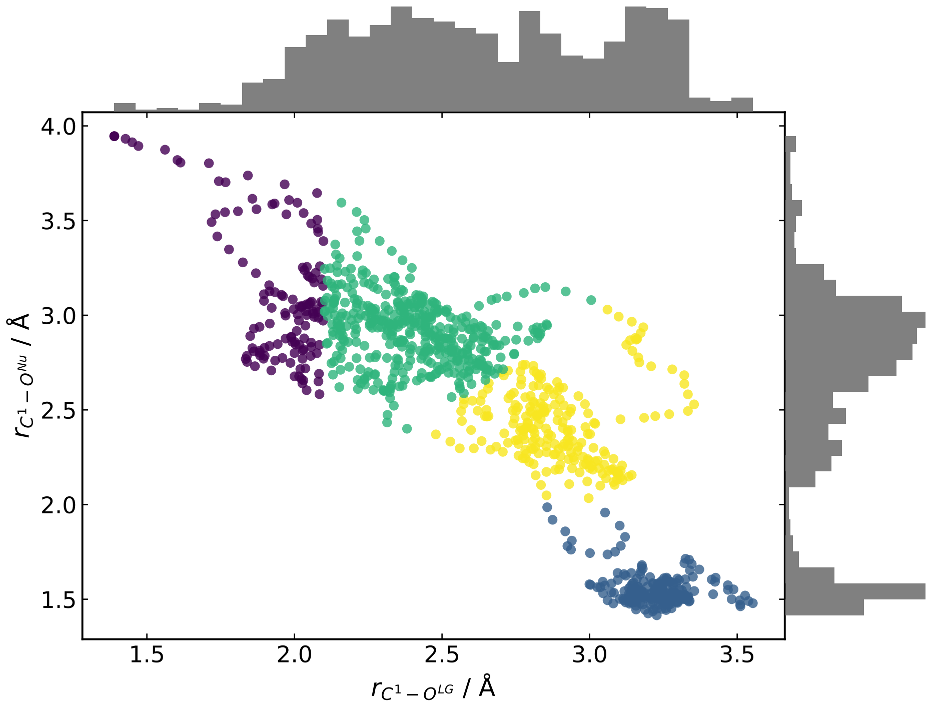

Figure S14: Distribution of testing data points generated from four uphill MD simulations for **R3**. The Histograms depict the distribution of bond lengths of $r_{\mathrm{C^1-O^{LG}}}$ (top) and $r_{\mathrm{C^1-O^{Nu}}}$(right). Data points are colour-coded into four categories: RS (131 configurations, purple), Inter1 (373 configurations, green), Inter2 (293 configurations, yellow), and PS (207 configurations, blue).

configurations in total), with six significant outliers, the largest having an absolute error of 2.11 meV atom^{-1}.

We then relabelled the 565 configurations collected in AL with energies and forces computed at the ωB97X-D3BJ/def2-TZVP level of theory, as this methodology is accurate in both energy barriers and geometries for organic reactions.[**omega-dft**] Additionally, the gas-phase substrate of six configurations, which exhibit the largest errors in the validation of MLIP with ground-truth of PBE-D3BJ/def2-TZVP, were also relabeled, resulting in a total of 571 configurations at the ωB97X-D3BJ/def2-TZVP level of theory.

The accuracy of the ACE MLIP was evaluated by comparing the energies and atomic forces at individual points between the ground-truth values and those predicted by the MLIP over a 1-ps MLIP-MD simulation initiated from RS and PS configurations (Figs. S16). The ACE MLIP exhibited energy errors of 0.85 and 0.46 meV atom^{-1} and forces errors of 21 and 17 meV Å^{-1} for each trajectory. The other two trajectories were generated by MLIP/MD, initiated from RS and PS respectively, and propagated for 1.5 ps with a time step of 0.5 fs. Across all 1,004 testing points coming from four independent trajectories, the energy and force errors were 0.60 meV atom^{-1} and 36.5 meV Å^{-1}, respectively. This error is comparable to the state-of-the-art system-specific neural-network potential, DeepMD, for the reaction of urea decomposition in explicit water, which has an energy error of 0.57 meV atom^{-1}. To ensure consistency with previous accuracy assessments and

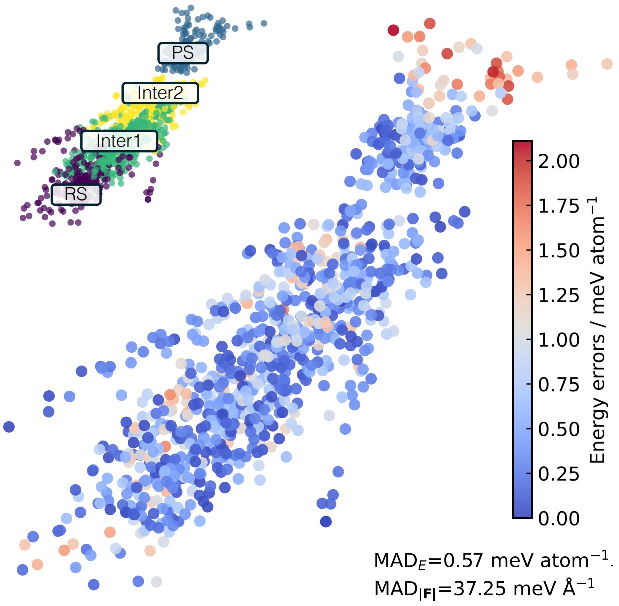

Figure S15: (top) Multidimensional scaling (MDS) map of the SOAP descriptor centred on anomeric centre, C^1, for 1,004 testing data points, coloured as RS, Inter1, Inter2, and PS. Inter1 and inter2 are determined by $r_{C^1-O^{LG}}$ and $r_{C^1-O^{Nu}}$. If $r_{C^1-O^{Nu}} > r_{C^1-O^{LG}}$, the configuration is assigned as Inter1; otherwise, it is considered Inter2. The same MDS map is also colour-coded based on energy error for MLIP prediction compared to the ground truth method (PBE-D3BJ/def2-TZVP), with high errors in red and low errors in blue.

to eliminate cumulative errors due to varying system sizes, energy errors were expressed in meV atom $^{-1}$ and force errors in meV Å^{-1}, aligning with the standards of the broader MLIP community. Furthermore, energy conservation in the dynamics and the stability of the potentials was confirmed by a 100-ps MLIP-MD simulation of the substrate immersed in 128 DCM molecules. Fig. S17 depicts the fluctuation in potential energy, ranging from -0.6 eV to 0.6 eV, while the total energy remains constant with minimal fluctuations over 100 ps, consistent with the NVE ensemble.

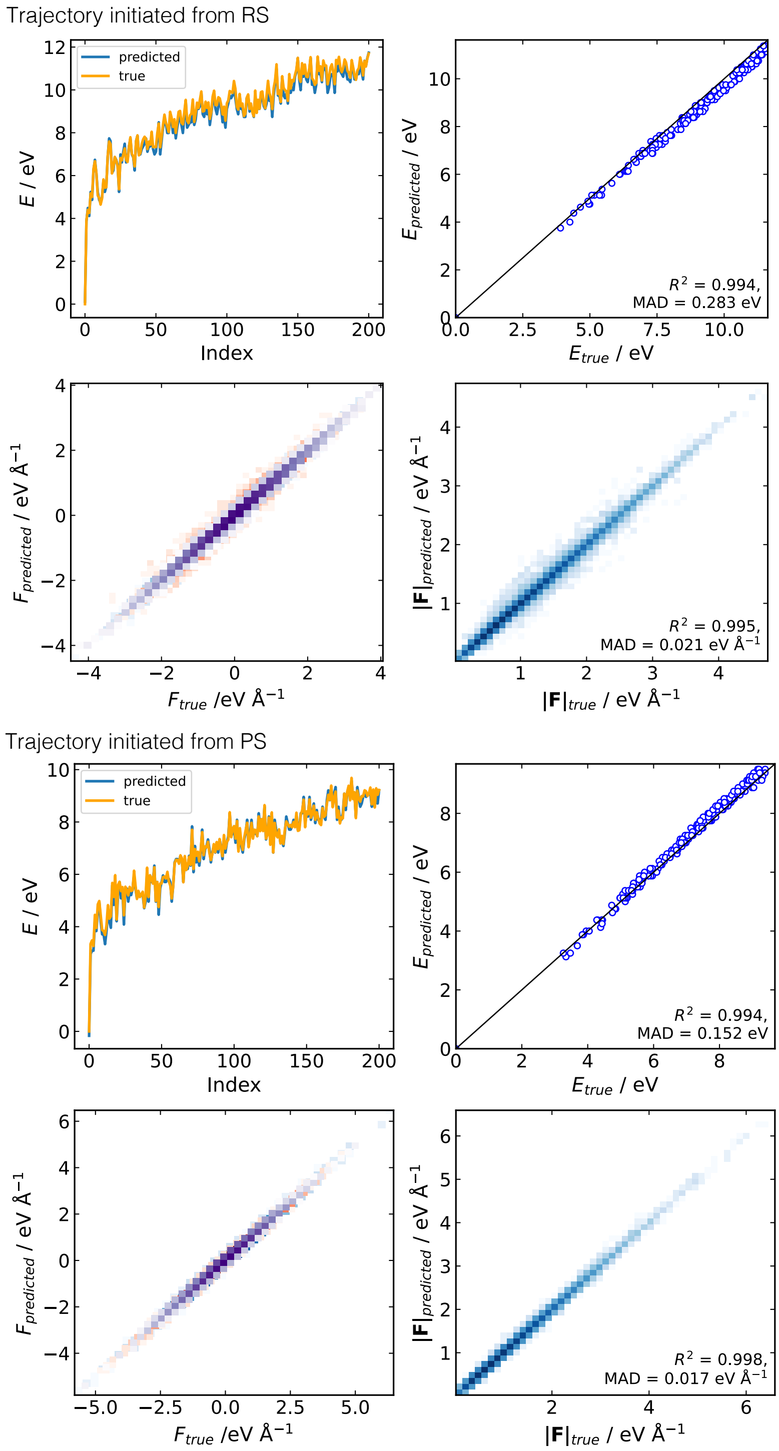

Figure S16: Comparison of ground-truth (ωB97X-D3BJ/def2-TZVP) and predicted (ACE MLIP) energies and forces for **R3** in DCM. Values were obtained from a 1-ps ACE MLIP-MD simulation (300 K, time step = 0.5 fs) initiated from (top) gas-phase optimised RS geometry immersed in 56 DCM molecules or (bottom) gas-phase optimised PS geometry. Prior to the MD simulation, solvent molecules were minimised while keeping the substrate geometry fixed.

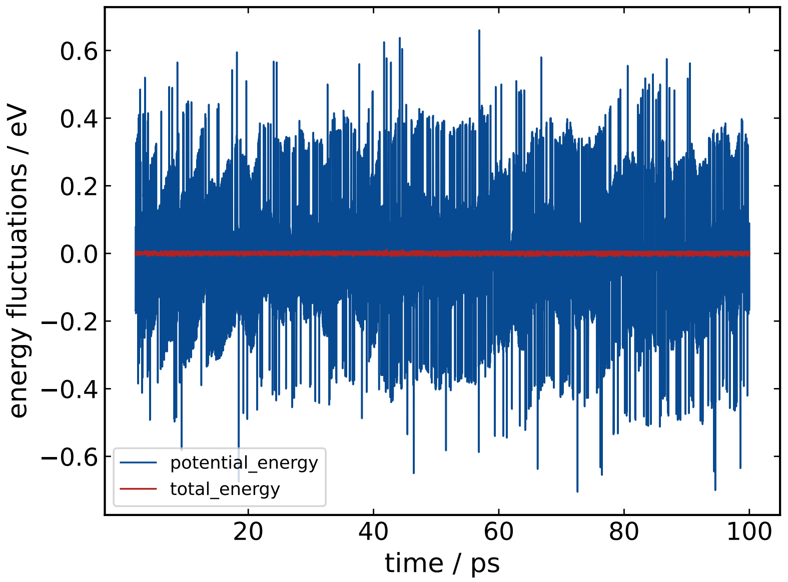

Figure S17: Energy fluctuations of the MLIP for the **R3** with 128 DCM during 100 ps NVE simulation under PBC

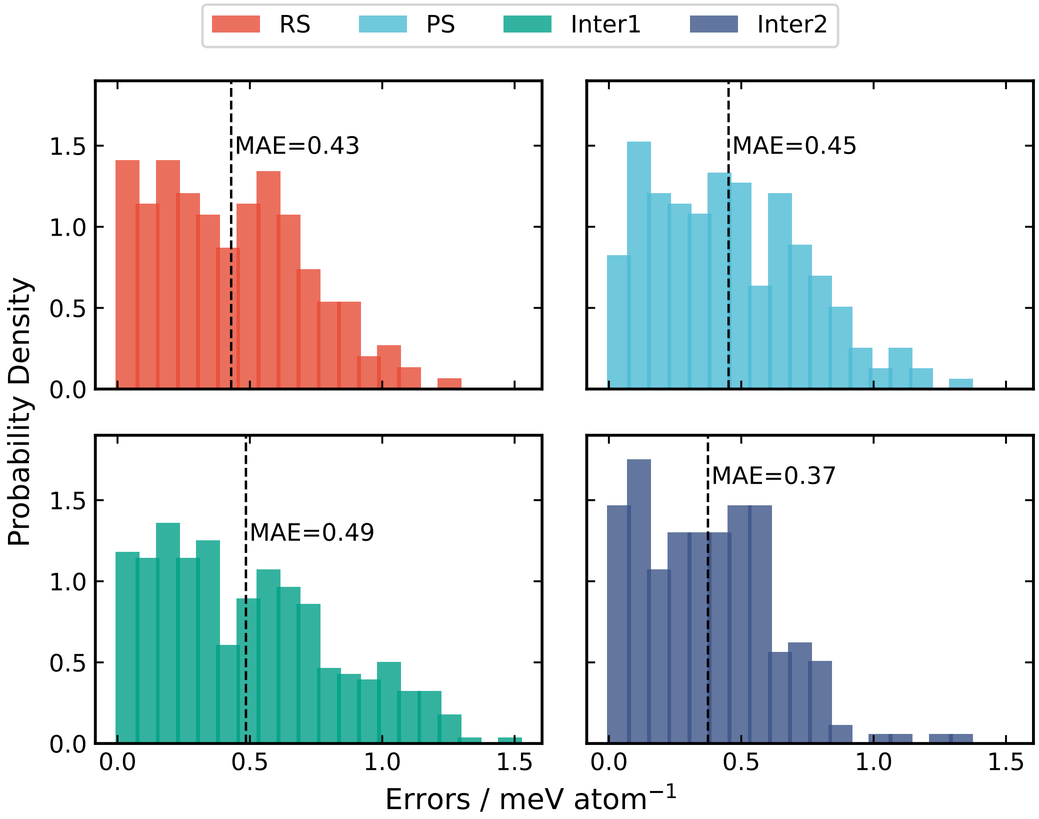

Figure S18: Distribution of MLIP energy prediction errors relative to the ground truth method (ωB97X-D3BJ/def2-TZVP) across four configuration classes: RS, Inter1, Inter2, and PS. The similar spread and location of the distributions indicate that there is no systematic correlation between energy error and configuration type, confirming that the MLIP maintains accuracy across the entire reaction pathway.

S4.2 Free energy calculations

The FES for **R3** was computed using WTMetaD/MLIP-MD, using MLIPs trained at both PBE-D3BJ/def2-TZVP and the ωB97X-D3BJ/def2-TZVP level of theory. Three WTMetaD/MLIP-MD simulations were conducted with different random seeds for both MLIPs, with coordination numbers (CN) as CVs, as detailed in the Computational Methods section of the main text. These simulations were performed in the NVT ensemble at 298 K for 300 ps with a time step of 0.5 fs, each maintaining stability throughout the entire duration.

S4.2.1 PBE-D3BJ/def-TZVP level of theory

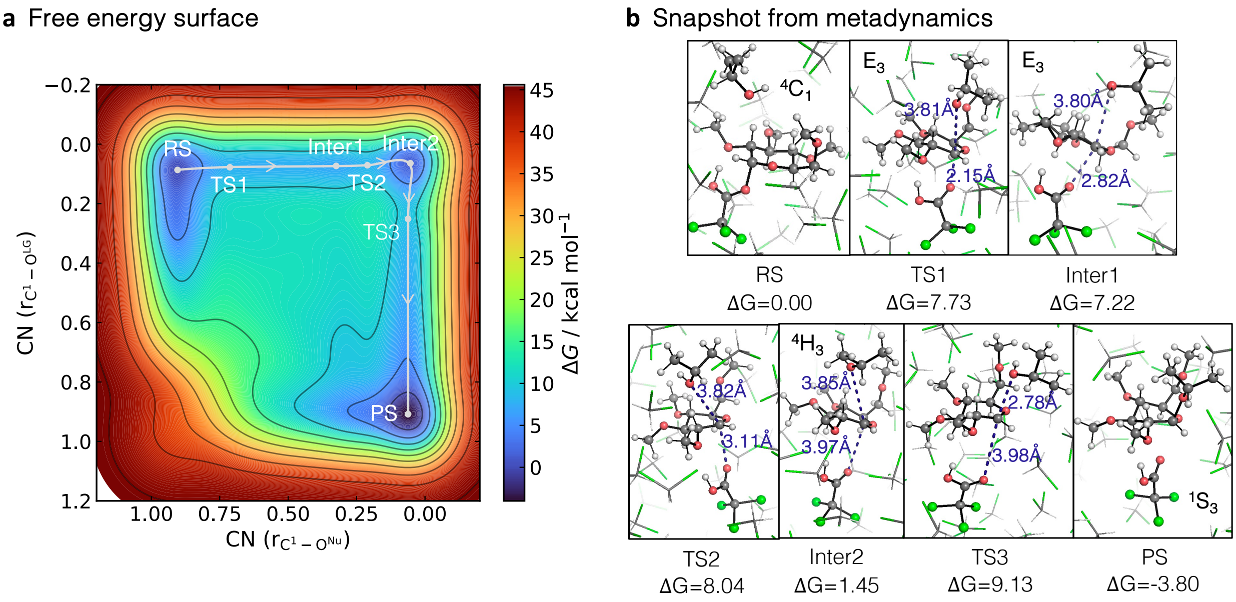

Figure S19: **a** Free energy surface of **R3** calculated by WTMetaD/MLP-MD along the collective variable (CV) of two coordination numbers (CN($r_{C^1-O^{LG}}$) and CN($r_{C^1-O^{LG}}$)) representing the breaking and forming bonds. The reaction pathway with critical points is highlighted. **b** Snapshots from metadynamics simulation illustrating RS, TSs intermediates (Inter) and PS. Key distances $r_{C^1-O^{LG}}$ and $r_{C^1-O^{Nu}}$, along with the corresponding free energy relative to RS, are highlighted.

The resulting free energy surface shows the reaction proceeds via a stepwise mechanism (Fig. S19a). TS1 corresponds to a dissociative TS with envelope (E_3)-like ion conformation characterized by bond lengths $r_{C^1-O^{LG}}$ of 2.15 Å and $r_{C^1-O^{Nu}}$ of 3.81 Å. This dissociative TS is consistent with the AIMD study [6], which reported $r_{C^1-O^{LG}}$ as 2.13 Å. After TS1, the $r_{C^1-O^{LG}}$ increases, leading to an intermediate state, Inter1, adopting an (E_3)-like ion conformation with $r_{C^1-O^{LG}}$ measuring 2.82 Å. Subsequently, TS2 links the (E_3)-like and half-chair ($^4H_3$)-like ion conformations (Inter2). This oxocarbenium Inter2 is 1.4 kcal mol^{-1}higher in energy than the RS (Fig. S19b).

TS3 characterized by $r_{C^1-O^{LG}} = 3.98$ Å and $r_{C^1-O^{Nu}} = 2.78$ Å (Fig. S19b). This TS corresponds to a conformational transition from a ($^4H_3$)-like (Inter2) to a skew-boat ($^1S_3$)-like (PS) structure, which is close to the one reported in AIMD study with $r_{C^1-O^{Nu}} = 2.66$ Å.[6] Following TS3, the

bond between the nucleophile and anomeric centre is formed. The barrierless nature of this step is corroborated by a geometry scan along $r_{C^1-O^{Nu}}$ from 3 Å to 1.5 Å, conducted at the ground-truth level of theory (PBE-D3BJ/def2-TZVP) with implicit solvent (Fig. S20), and has been observed in other glycosylation reactions.[7]

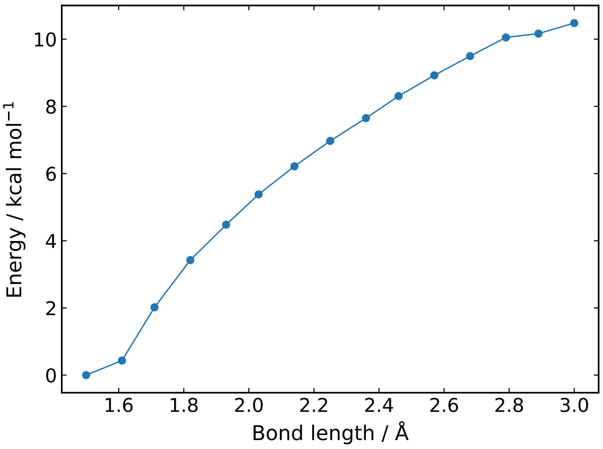

Figure S20: Energy scan along the $r_{C^1-O^{Nu}}$ from 3.0 Å to 1.5 Åcomputed at the PBE-D3BJ/def2-TZVP level of theory.

Overall, the mechanism of **R3** follows a stepwise S$_N$i pathway, consistent with previously published AIMD simulations for a similar system in DCM.[6] This involves an initial dissociative TS (TS1) leading to a conformations change to form an oxocarbenium intermediate (Inter2), followed by conformational change resulting in a barrierless nucleophilic attack step forming PS (Fig. S19a).

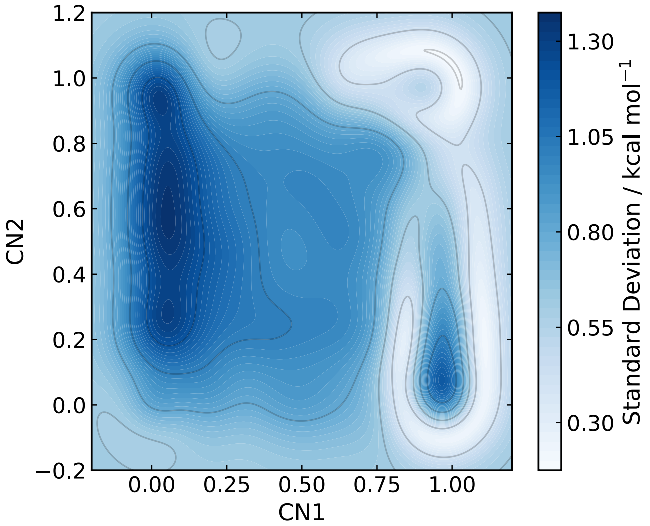

Figure S21: Free energy standard deviations for three runs of WTMetaD/MLP-MD.

To validate the convergence of WTMetaD simulations, we calculated the standard deviation of the FES from three independent runs, as illustrated in Fig. S21. The maximum standard deviation

observed in the FES is 1.32 kcal mol^{-1}, indicating the convergence and reliability of the FES obtained via WTMetaD/MLP-MD. Additionally, block averaging analysis of the standard deviation over the final 200 ps of the trajectory from a stable run, depicted in Fig. S22a, corroborates this convergence. The error stabilises at a block size of approximately 1500, with a value of 0.1 kcal mol^{-1}. Fig. S22b illustrates the bond length changes during 300 ps WTMetaD trajectory, which confirms that several chemical processes occur during the sampling.

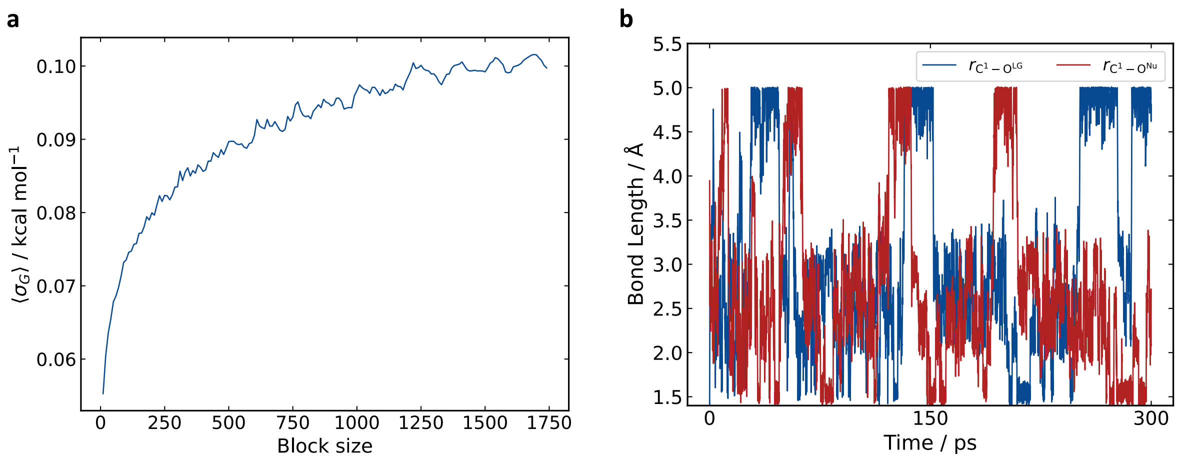

Figure S22: (a) Block averaging analysis on the standard deviation for the free energy of WTMetaD/MLP-MD simulations on final 200 ps trajectory for the reaction **R3**. (b) Changes of $r_{C^1-O^{LG}}$ and $r_{C^1-O^{Nu}}$ along a 300-ps WTMetaD trajectory

The computational cost of AIMD highlights the advantage of MLIP. A single-point gradient calculation for a system consisting of the substrate in 86 DCM using the PBE-D3BJ/def2-TZVP level of theory takes approximately 11 hours with 8 CPUs. Since WTMetaD/AIMD simulations require gradient calculations at each MD step, propagating 300 ps would take over 750 years ($11 \times 600000 = 763.89$ years) on the same hardware. In constrast, training the MLIP with ground-truth of PBE-D3BJ/def2-TZVP requires approximately 2390 CPU hours. Specifically, Subsets 1, 2, and 3 account for about 270, 1640, and 470 CPU hours, respectively. Additionally, three repeated MetaD calculations for FES require approximately 3456 CPU hours. This computational demand could be further reduced by implementing a neural network-based MLIP with GPU acceleration. The total computational cost for MLIP, encompassing both training and FES calculations, is equivalent to the computational requirement for 66 steps and 33 femtoseconds of metadynamics driven by AIMD.

S4.2.2 ωB97X-D3BJ/def2-TZVP level of theory

To validate the convergence of WTMetaD simulations, we calculated the standard deviation of the FES across three independent runs (Fig. S23). The maximum observed standard deviation of 2.35 kcal mol^{-1}confirms the convergence and reliability of the FES obtained via WTMetaD/MLIP-MD. Additionally, block averaging analysis over the final 200 ps of the trajectory from a trajectory (Fig.

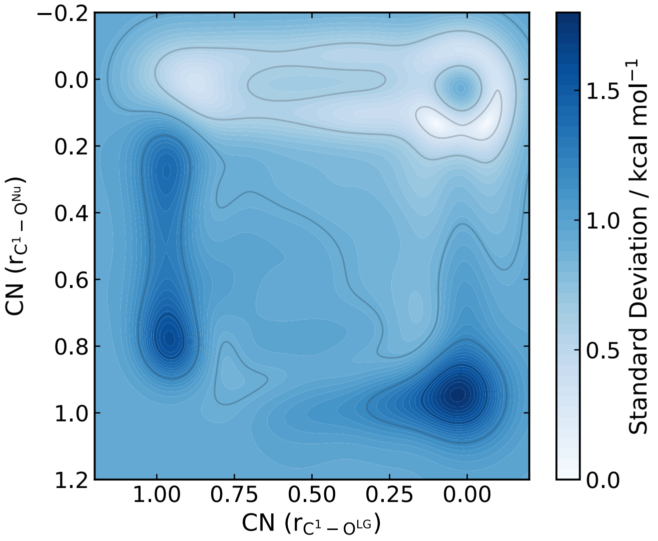

Figure S23: Free energy standard deviations (in kcal mol^{-1}) for three runs of WTMetaD/MLIP-MD.

S24a) further supports this convergence, with the error stabilising at a block size of approximately 1250 and a value of 0.055 kcal mol^{-1}. Fig. S24b shows the root mean square difference between FESs as a function of time, reaching a zero plateau after 200 ps, indicating the simulation time for FES is long enough to converge. In the FES calculations, the intermediate has a lower energy compared to the RS. It is important to note, the RS referred here is an acid-activated RS, where the leaving group is protonated. This structure is 13.5 kcal mol^{-1} higher in energy compared to the neutral RS in complex with activator $HNTf_2$. The energy was calculated at CPCM(DCM)-ωB97X-D3BJ/def2-TZVP level of theory. The structures of protonated and neutral complexes are available in the figshare repository.

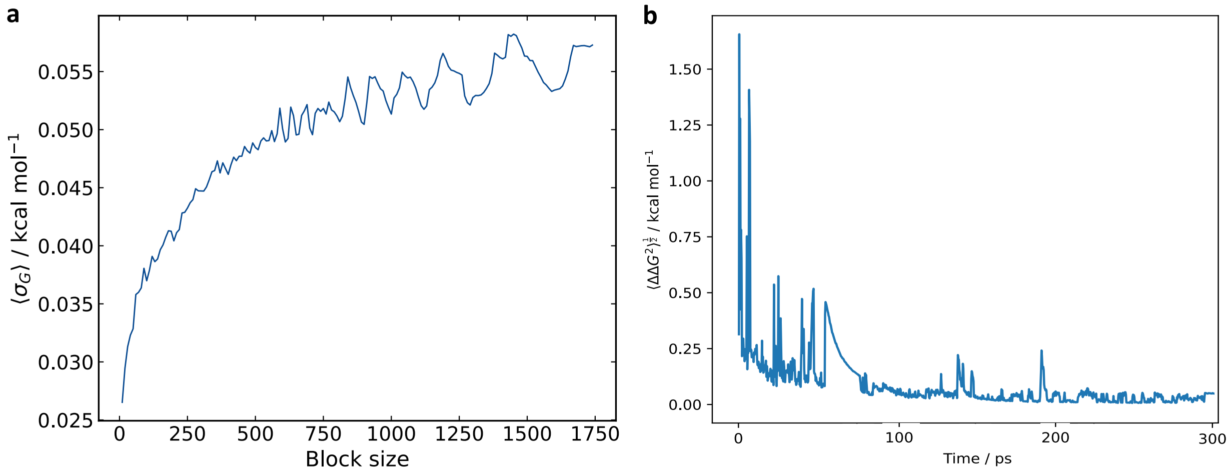

Figure S24: (a) Block averaging analysis of the standard deviation for the free energy over the final 200 ps trajectory of WTMetaD/MLIP-MD simulations for **R3**. (b) Root mean square difference between free energy surfaces as a function of time

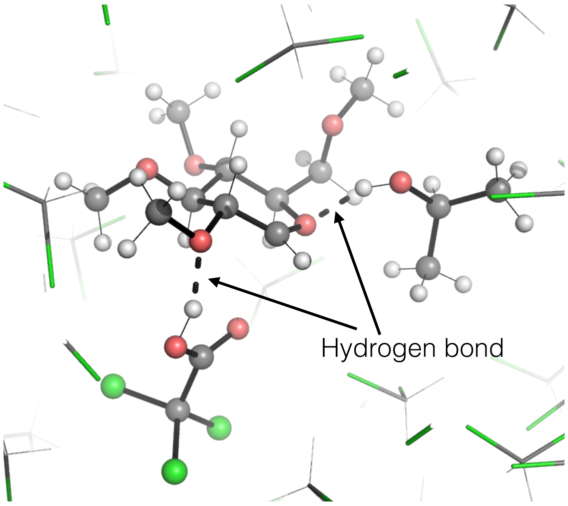

Figure S25: Snapshot of an intermediate from a trajectory initiated from Inter2, highlighting the hydrogen bonds between the leaving group and the oxocarbenium ion, as well as between the nucleophilic group and the oxocarbenium ion.

S4.3 Accuracy of MLIP with chloride counterion

To further evaluate the extrapolation capabilities of the resulting MLIP, we selected an additional system consisting of the substrate, 54 DCM molecules, and one chloride ion within a 19.6 Å box (Fig. S26). The initial configuration of this test set was generated by placing the gas-phase RS within 54 DCM solvent molecules. The substrate was then placed at the centre of the box, while chloride was positioned at the corner of the box. Solvent molecules overlapping with the substrate or chloride were removed. This configuration was then used to run 1 ps MLIP-MD trajectory, following BFGS optimisation, with a fixed substrate position constraint applied in MLIP-MD and minimization steps. All simulations were performed with PBC. The accuracy of the MLIP was confirmed by a point-to-point comparison between the ground-truth (PBE0-D3BJ/def2-SVP) and MLIP-predicted energies and forces along the trajectory (Fig. S26), with an energy error of 81.2 meV atom^{-1} and a force error of 44 meV Å^{-1}. Although the energy error is nearly double that of testing sets without a counterion (0.57 meV atom^{-1}), the force error remains comparable (37.25 meV Å^{-1}). Therefore, the MLIP shows accuracy for systems containing counterions despite the absence of counterion information in the training data, highlighting the extrapolation capabilities of the resulting MLIP.

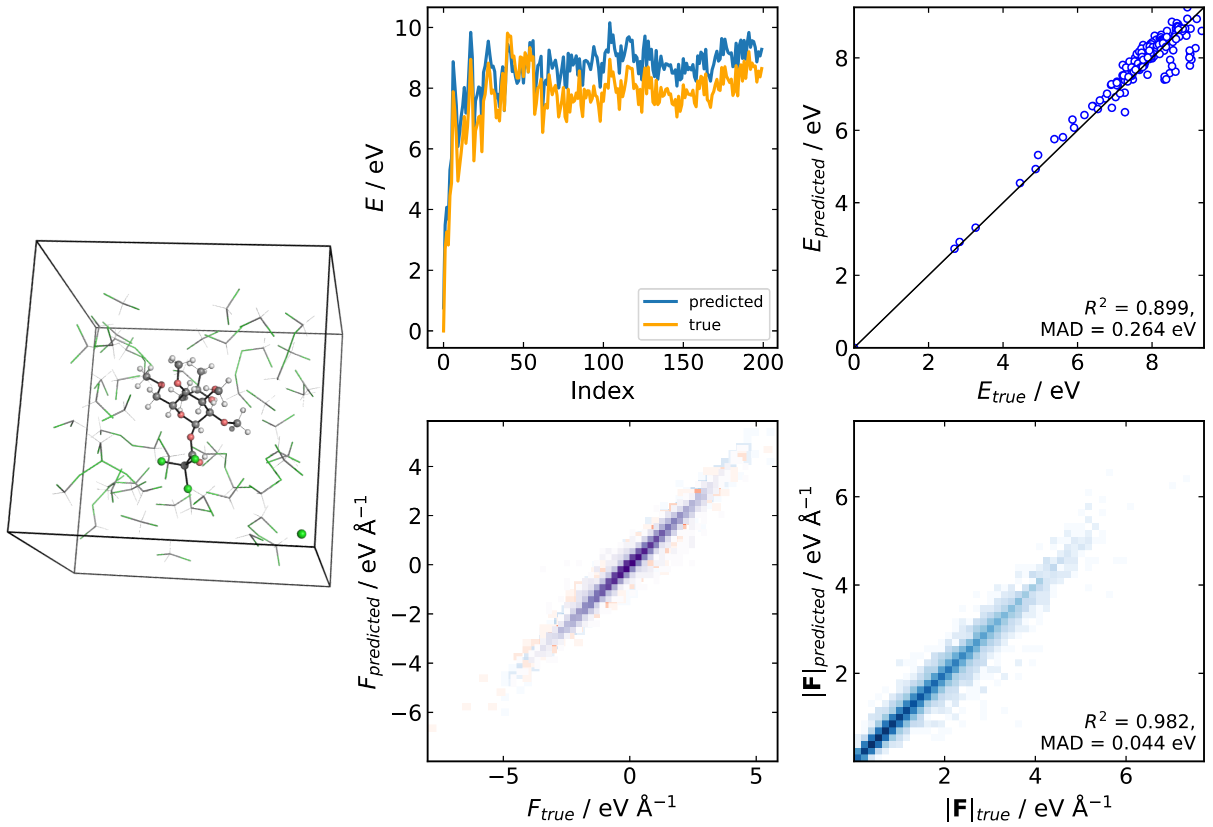

Figure S26: Comparisons of ground-truth (PBE0-D3BJ/def2-SVP) and predicted (ACE MLIP) energies and forces for **R3** in DCM with chloride. Values obtained during 1-ps ACE MLIP-MD trajectory initiated from the gas-phase RS geometry immersed in 54 DCM molecules and one chloride(300 K, time step = 0.5 fs).

References

(1) Young, T. A.; Johnston-Wood, T.; Zhang, H. W.; Duarte, F. *Phys. Chem. Chem. Phys.* **2022**, *24*, 20820–20827.

(2) Zhang, H.; Juraskova, V.; Duarte, F. *Nat. Commun* **2024**, *15*, 6114.

(3) Barducci, A.; Bussi, G.; Parrinello, M. *Phys. Rev. Lett.* **2008**, *100*, 020603.

(4) Bussi, G.; Tribello, G. A. *Biomolecular Simulations: Methods and Protocols* **2019**, *2022*, 529–578.

(5) Bartok, A. P.; Kondor, R.; Csanyi, G. *Phys. Rev. B* **2013**, *87*, 184115.

(6) Fu, Y.; Bernasconi, L.; Liu, P. *J. Am. Chem. Soc.* **2021**, *143*, 1577–1589.

(7) Santana, A. G.; Montalvillo-Jiménez, L.; Díaz-Casado, L.; Corzana, F.; Merino, P.; Cañada, F. J.; Jiménez-Osés, G.; Jiménez-Barbero, J.; Gómez, A. M.; Asensio, J. L. *J. Am. Chem. Soc.* **2020**, *142*, 12501–12514.
